# Biomarker panels associated with progression of renal disease in type 1 diabetes

**DOI:** 10.1007/s00125-019-4915-0

**Published:** 2019-06-20

**Authors:** Marco Colombo, Erkka Valo, Stuart J. McGurnaghan, Niina Sandholm, Luke A. K. Blackbourn, R. Neil Dalton, David Dunger, Per-Henrik Groop, Paul M. McKeigue, Carol Forsblom, Helen M. Colhoun, on behalf of the FinnDiane Study Group and the Scottish Diabetes Research Network (SDRN) Type 1 Bioresource Collaboration

**Affiliations:** 1 Usher Institute of Population Health Sciences and Informatics, University of Edinburgh, Edinburgh, UK; 2 Folkhälsan Institute of Genetics, Folkhälsan Research Center, Helsinki, Finland; 3 Abdominal Center Nephrology, University of Helsinki and Helsinki University Hospital, Helsinki, Finland; 4 Research Program for Clinical and Molecular Metabolism, Faculty of Medicine, University of Helsinki, Helsinki, Finland; 5 MRC Institute of Genetics and Molecular Medicine, University of Edinburgh, Western General Hospital, Crewe Road South, Edinburgh, EH4 2XU UK; 6 WellChild Laboratory, Evelina London Children’s Hospital, Guy’s and St Thomas’ National Health Service Foundation Trust, London, UK; 7 Department of Paediatrics, University of Cambridge, Cambridge, UK; 8 Wellcome Trust-MRC Institute of Metabolic Science, University of Cambridge, Cambridge, UK; 9 Department of Diabetes, Central Clinical School, Monash University, Melbourne, VIC Australia; 10 Public Health, NHS Fife, Kirkcaldy, UK

**Keywords:** Clinical science, Epidemiology, Metabolomics, Nephropathy, Proteomics

## Abstract

**Aims/hypothesis:**

We aimed to identify a sparse panel of biomarkers for improving the prediction of renal disease progression in type 1 diabetes.

**Methods:**

We considered 859 individuals recruited from the Scottish Diabetes Research Network Type 1 Bioresource (SDRNT1BIO) and 315 individuals from the Finnish Diabetic Nephropathy (FinnDiane) study. All had an entry eGFR between 30 and 75 ml min^−1^[1.73 m]^−2^, with those from FinnDiane being oversampled for albuminuria. A total of 297 circulating biomarkers (30 proteins, 121 metabolites, 146 tryptic peptides) were measured in non-fasting serum samples using the Luminex platform and LC electrospray tandem MS (LC-MS/MS). We investigated associations with final eGFR adjusted for baseline eGFR and with rapid progression (a loss of more than 3 ml min^−1^[1.73 m]^−2^ year^−1^) using linear and logistic regression models. Panels of biomarkers were identified using a penalised Bayesian approach, and their performance was evaluated through 10-fold cross-validation and compared with using clinical record data alone.

**Results:**

For final eGFR, 16 proteins and 30 metabolites or tryptic peptides showed significant association in SDRNT1BIO, and nine proteins and five metabolites or tryptic peptides in FinnDiane, beyond age, sex, diabetes duration, study day eGFR and length of follow-up (all at *p* < 10^−4^). The strongest associations were with CD27 antigen (CD27), kidney injury molecule 1 (KIM-1) and α1-microglobulin. Including the Luminex biomarkers on top of baseline covariates increased the *r*^2^ for prediction of final eGFR from 0.47 to 0.58 in SDRNT1BIO and from 0.33 to 0.48 in FinnDiane. At least 75% of the increment in *r*^2^ was attributable to CD27 and KIM-1. However, using the weighted average of historical eGFR gave similar performance to biomarkers. The LC-MS/MS platform performed less well.

**Conclusions/interpretation:**

Among a large set of associated biomarkers, a sparse panel of just CD27 and KIM-1 contains most of the predictive information for eGFR progression. The increment in prediction beyond clinical data was modest but potentially useful for oversampling individuals with rapid disease progression into clinical trials, especially where there is little information on prior eGFR trajectories.

**Electronic supplementary material:**

The online version of this article (10.1007/s00125-019-4915-0) contains peer-reviewed but unedited supplementary material, which is available to authorised users.

## Introduction

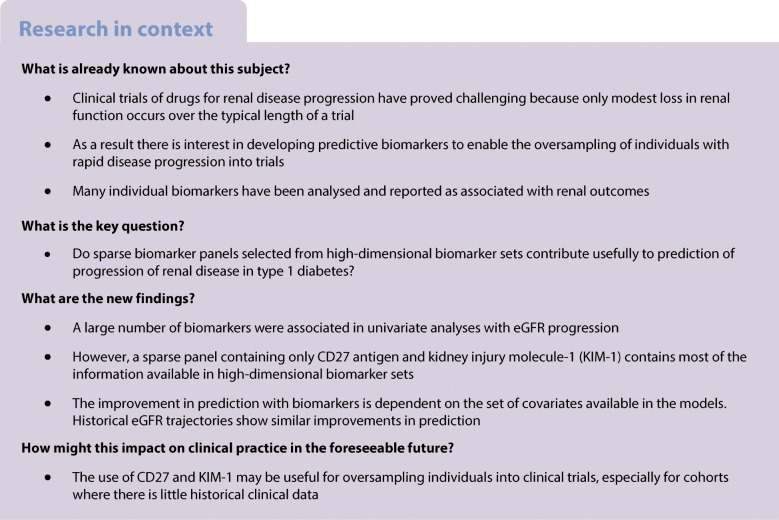



Diabetic kidney disease (DKD) is the cause of most renal failure and impaired renal function in type 1 diabetes mellitus. As such it is a major contributor to the reduced life span in type 1 diabetes [1]. Accordingly, developing drugs to prevent or reverse impaired renal function is an important goal. However, there is wide variation in the rate of renal function decline among those with type 1 diabetes with some people being much more susceptible than others. This makes conducting clinical trials of drugs challenging because, over the typical trial follow-up time, the average loss in renal function is modest [2]. An improved ability to predict which people with diabetes will progress most rapidly would facilitate oversampling of such people into clinical trials, thereby improving trial power.

To this end there have been several large-scale investments into developing biomarkers for renal disease progression in diabetes. However, as we recently reviewed [3], while there are many sporadic reports of single biomarkers, there have been few systematic attempts to harness the potential of high-dimensional biomarker assays for prediction. Furthermore, many studies do not evaluate how well biomarkers improve prediction on top of clinical record data.

In this study we aimed to identify circulating serum biomarkers that are associated with progression of renal disease in individuals with type 1 diabetes mellitus and to assess their usefulness in a prediction setting when either limited or comprehensive sets of clinical characteristics are available. We evaluated the same set of biomarkers in two different cohorts, the Scottish Diabetes Research Network Type 1 Bioresource (SDRNT1BIO) [4] and the Finnish Diabetic Nephropathy study (FinnDiane) [5], to assess reproducibility and generalisability of the results across a range of characteristics. Moreover, we used two different technologies (Luminex and LC electrospray tandem MS [LC-MS/MS]) to obtain a comprehensive coverage of both candidate and discovery biomarkers for renal disease.

The objectives of this study were first, to determine the association of each biomarker measured with eGFR achieved at the end of follow-up and with rapid progression status in both cohorts; and second, to select from these a parsimonious panel of biomarkers that would be helpful in predicting renal function decline in both cohorts.

## Methods

### Participants

SDRNT1BIO [4] is a prospective cohort study comprising 6127 people with a clinical diagnosis of type 1 diabetes mellitus, representing 25% of all adults with type 1 diabetes in Scotland, recruited between December 2010 and November 2013. At recruitment, clinical measurements and a blood sample were taken. From electronic healthcare records we extracted routine health-related data at biosample date, or the closest up to 18 months before. These were supplemented by direct measures of eGFR and albuminuria from samples collected at recruitment. For this study we selected 859 individuals with eGFR between 30 and 75 ml min^−1^ [1.73 m]^−2^ at study biosample date and with at least three prospective eGFR determinations over a period of at least 2 years or incident end-stage renal disease (ESRD).

The FinnDiane study is a prospective nationwide multicentre study comprising more than 8400 adults with type 1 diabetes mellitus covering more than 15% of all the type 1 diabetic individuals in Finland [5]. Individuals participated in the study during a regular visit to their attending physician during which detailed demographic and medical history data were collected with standardised questionnaires. For this study, a subpopulation of 315 participants studied between 1994 and 2011 was included, comprising those who at biosample date had an eGFR between 30 and 75 ml min^−1^ [1.73 m]^−2^ and who had micro- or macroalbuminuria. Participants were also required to have an eGFR measurement within half a year from the biosample date and at least three prospective eGFR determinations over a time period of at least 3 years. If the person developed ESRD, three prospective eGFR measurements over a time period of at least 1 year was accepted.

In both studies participants were classified as normo-, micro- or macroalbuminuric at baseline according to their albumin/creatinine ratio (ACR) falling in the intervals 0–3.39 mg/mmol, 3.39–33.9 mg/mmol, or above 33.9 mg/mmol, based on two out of three consecutive measurements before baseline.

Both studies were performed in accordance with the Declaration of Helsinki; all participants gave their written consent and the study protocol was approved by the local ethics committees.

### Renal outcomes

eGFR was calculated with the CKD-EPI equation [6] using serum and plasma creatinine values retrieved retrospectively and prospectively from medical records. In SDRNT1BIO, these excluded readings concurrent with hospital admissions.

A summary measure of the historical eGFR was obtained by computing a weighted average of all retrospective eGFR records for each person, with weights inversely related to the amount of time leading to the biosample date. Participants with no retrospective eGFR data had their historical eGFR imputed to study day eGFR.

Achieved eGFR was defined as the median eGFR reading of the last 6 months of follow-up. Initiation of renal replacement therapy (RRT) was considered to indicate an achieved eGFR of 10 ml min^−1^ [1.73 m]^−2^ and all subsequent readings were censored.

The decline of renal function was estimated by fitting a simple linear regression model to the serial prospective eGFR determinations of each person. A rapid progressors category was defined by dichotomising prospective linear slopes according to a threshold of an average loss of more than 3 ml min^−1^ [1.73 m]^−2^ year^−1^.

### Biomarkers measured and analysed

We measured a total of 297 biomarkers in non-fasting serum samples using: (1) the Luminex platform at the CLIA certified Myriad RBM laboratory (Austin, Texas, USA) to assay 30 protein biomarkers; and (2) LC-MS/MS at the WellChild laboratory (King’s College London, UK) to capture 121 metabolites (including six ratios between pairs of metabolites) and 146 tryptic peptides.

A number of quality control steps were performed in each of the two studies prior to the statistical analysis stage. This led to excluding from the analyses biomarkers considered uninformative: those that reported over 98% of undetectable readings and those that had more than 50% of their values missing at random (19 and 8 in SDRNT1BIO, and 20 and 7 in FinnDiane, respectively). We evaluated reproducibility of biomarker measurements by computing intra-class correlations on duplicate samples from a pilot study on a subset of participants (35 duplicate samples in SDRNT1BIO, 25 in FinnDiane). According to this, we removed biomarkers that had an intra-class correlation <0.4 (41 in SDRNT1BIO and 59 in FinnDiane).

Serum samples were stored at −80°C in SDRNT1BIO and −20°C in FinnDiane. Multiple freeze/thaw cycles and suboptimal storage conditions are known to affect some biomarkers. In a separate study we considered 16 FinnDiane sample aliquots stored at −20°C and −80°C: this allowed us to identify which biomarkers are susceptible to, and which are unaffected by, poor storage conditions. Among the biomarkers that passed the quality control steps above, 24 biomarkers were identified to be affected by storage conditions and removed from further analyses in FinnDiane, while they were still considered for SDRNT1BIO in univariate analyses.

Overall, 191 biomarkers were analysed in SDRNT1BIO (27 proteins, 81 metabolites and ratios, and 83 tryptic peptides) and 167 biomarkers in FinnDiane (22 proteins, 63 metabolites and ratios, and 82 tryptic peptides).

Within each study, left-censored values were imputed to half the detection threshold; right-censored values (only present for N-terminal prohormone of brain natriuretic peptide) were imputed to the largest value reported; values missing at random were imputed to the median.

### Univariate analysis

Biomarkers were evaluated independently in linear and logistic regression models adjusted for age, sex, diabetes duration, eGFR at biosample date and length of follow-up (basic covariates). To incorporate more information about past and current status of renal function, we also considered models containing ACR category and the weighted average of historical eGFR and alongside the basic set of covariates. We further adjusted models for BMI, systolic BP (SBP), diastolic BP (DBP), HbA_1c_, HDL-cholesterol, total cholesterol, smoking status, ACR category and weighted average of historical eGFR (full covariates). All continuous variables were Gaussianised prior to fitting the models, and standardised to zero mean and unit SD. Associations were declared significant at *p* < 10^−4^, which is particularly conservative even for the number of tests performed (Bonferroni-corrected threshold would be 0.05/191 = 2.6 × 10^−4^).

### Construction of sparse panels of biomarkers

Only the biomarkers with valid data in both studies were considered for the purpose of the selection of a sparse panel of biomarkers (22 from the Luminex platform and 145 from the LC-MS/MS platform). The two biomarker sets were modelled independently from each other. However, for comparison we also generated panels of biomarkers using both platforms simultaneously, although a panel requiring two platforms would not be cost effective or easily deployable in the clinical setting.

We adopted a Bayesian modelling approach based on hierarchical shrinkage priors [7], in which the clinical covariates used to control for confounding in the models were assigned a Gaussian prior (which induces some shrinkage), while the biomarkers were penalised through the horseshoe prior to promote sparsity [8, 9]. The hierarchical shrinkage approach was implemented using the Stan Bayesian inference framework [10]. See electronic supplementary material (ESM) Methods for more details.

We evaluated the predictive performance of the biomarker models on withdrawn data using 10-fold cross-validation, and compared models including biomarkers to baseline (unpenalised) models that only contain clinical covariates, also fitted using Stan. For each set of baseline covariates used, we reported the difference in log-likelihood (computed on the test observations from 10-fold cross-validation, and expressed in natural log units) between the model with biomarkers and the model including only the clinical covariates. For linear regression models we computed the *r*^2^ as the squared Pearson correlation coefficient between observed and predicted outcome. For logistic regression models, besides reporting the area under the receiver operating characteristic curve (AUC), we also presented the expected information for discrimination *Λ* expressed in bits [11]. This is a better measure of the incremental contribution of biomarkers to the predictive performance, as it captures the amount of additional information that they contain over and beyond the initial set of clinical covariates (see ESM Methods for more details). Computations were done with the R package wevid (version 0.6: https://CRAN.R-project.org/package=wevid).

To recover a sparse model, we then applied a projection approach according to which the high-dimensional posterior draws of the model containing all biomarkers (full model) are projected to lower-dimensional subspaces [12, 13] (see ESM Methods for more details). This procedure allowed us to rank the biomarkers in terms of importance. Each candidate model was then evaluated in terms of their contribution to the predictive performance relative to the performance of the full model, so that we could plot the relative explanatory power obtained by biomarker panels of different sizes.

## Results

### Participant characteristics

Table 1 reports the summary characteristics for the two cohorts analysed.

**Table 1:** Cohort characteristics at baseline

Covariate	SDRNT1BIO (*n* = 859)		FinnDiane (*n* = 315)		*p* value^a^
	Frequency/Median (IQR)	MaR	Frequency/Median (IQR)	MaR	
Age (years)	55.5 (46.1, 64.4)	0	46.3 (36.6, 52.5)	0	<1 × 10^−16^
Sex (female) (%)	56.8	0	44.8	0	2 × 10^−4^
Diabetes duration (years)	26.5 (17.4, 37.5)	0	31.9 (25.4, 37.9)	0	2 × 10^−12^
Length of follow-up (years)	5.2 (4.4, 5.7)	0	8.8 (5.9, 12.2)	0	<1 × 10^−16^
Start of follow-up (calendar year)	2012 (2011, 2012)	0	1999 (1998, 2001)	0	–
End of follow-up (calendar year)	2017 (2017, 2017)	0	2010 (2006, 2013)	0	–
eGFR (ml min^−1^ [1.73 m]^−2^)	72.0 (62.1, 84.5)	0	58.6 (45.7, 67.0)	0	<1 × 10^−16^
Achieved eGFR (ml min^−1^ [1.73 m]^−2^)	73.1 (58.6, 85.2)	0	29.9 (10.0, 55.9)	0	<1 × 10^−16^
Weighted average of historical eGFR (ml min^−1^ [1.73 m]^−2^)	81.2 (70.6, 91.4)	32	64.8 (52.4, 76.7)	108	<1 × 10^−16^
ACR (mg/mmol)	0.5 (0.3, 1.9)	21	25.0 (7.6, 69.5)	56	8 × 10^−15^
ACR category (normo/micro/macro)^b^ (%)	81.4/10.5/5.7	21	0/27.3/72.7	0	<1 × 10^−16^
Prospective eGFR slope (ml min^−1^ [1.73 m]^−2^ year^−1^)	−0.8 (−2.8, 0.7)	0	−2.4 (−4.4, −1.0)	0	3 × 10^−13^
Rapid progressors (slope < −3) (%)	22.6	0	40.3	0	2 × 10^−09^
HbA_1c_ (mmol/mol)	68.0 (60.0, 79.0)	0	71.6 (62.8, 82.5)	2	1 × 10^−02^
HbA_1c_ (%)	8.4 (7.6, 9.4)	0	8.7 (7.9, 9.7)	2	1 × 10^−02^
BMI (kg/m^2^)	27.2 (24.6, 30.5)	3	25.7 (23.2, 28.2)	2	1 × 10^−09^
HDL-cholesterol (mmol/l)	1.6 (1.3, 1.9)	19	0.9 (0.7, 1.1)	0	<1 × 10^−16^
Total cholesterol (mmol/l)	4.5 (3.9, 5.1)	6	5.1 (4.5, 5.7)	0	<1 × 10^−16^
SBP (mmHg)	134.0 (122.0, 146.0)	0	144.5 (131.0, 158.0)	4	6 × 10^−14^
DBP (mmHg)	74.0 (68.0, 80.0)	0	80.0 (72.5, 89.0)	4	<1 × 10^−16^
Ever smoker (%)	64.5	0	52.1	23	1 × 10^−02^
On any anti-hypertensive treatment (%)	61.7	0	94.3	3	<1 × 10^−16^
On ACE or ARB (%)	56.5	0	86.0	3	<1 × 10^−16^

We report median and IQR for continuous variables, and frequency for categorical variables

^a^*p* value is for the difference in means or proportions between the two cohorts

^b^For the ACR category we compared normoalbuminuric to all others

ARB, angiotensin II receptor blocker; MaR, number of observations missing at random

The length of follow-up was shorter in SDRNT1BIO as compared with FinnDiane (5.2 vs 8.8 years), the former being a more recently established cohort. FinnDiane participants were generally at a more advanced stage of renal function decline, with starting eGFR being lower despite their younger age, reflecting the fact that these individuals were oversampled for albuminuria. Similarly, the rate of progression of renal decline detectable during follow-up differed between the two cohorts in terms of prospective eGFR slopes (−0.83 vs −2.44 ml min^−1^ [1.73 m]^−2^ year^−1^ in SDRNT1BIO and FinnDiane, respectively) and of rapid progression (22.6% vs 40.3%). ESM Table 1 shows the characteristics of rapid progressors to non-progressors in each cohort. Of note, point estimates for HbA_1c_ and SBP are somewhat higher, and HDL-cholesterol lower, in progressors than non-progressors in both cohorts.

### Biomarkers explored

ESM Table 2 shows the full list of biomarkers measured with median, interquartile range (IQR) and range in each of the studies, and reason for removal of a biomarker from the analysis. There are important distributional differences in some of the biomarkers that may be due to depletion caused by suboptimal storage conditions of the FinnDiane samples, and may also reflect the more advanced stage of kidney disease in FinnDiane.

### Univariate associations

When modelling achieved eGFR adjusted for age, sex, diabetes duration, eGFR and length of follow-up, 46 and 14 biomarkers were statistically significant in SDRNT1BIO and FinnDiane, respectively, and 12 were significant in both. Table 2 shows remarkable consistency in the strongest associations between the two cohorts, with CD27 antigen (CD27) having the largest effect size in both studies. Effect sizes in FinnDiane, where albuminuria rates were higher, were generally larger than in SDRNT1BIO. Consistent with this, there was some evidence that associations were stronger in those with above rather than below median ACR in SDRNT1BIO (e.g. for CD27: β = −0.31 vs −0.21, *p* = 0.021 for interaction).

**Table 2:** Associations of each biomarker (considered separately) with achieved eGFR from linear regression models adjusted for age, sex, duration of diabetes, study day eGFR and length of follow-up

Biomarker	SDRNT1BIO		FinnDiane	
	β coefficient (95% CI)	*p* value	β coefficient (95% CI)	*p* value
Luminex proteins				
CD27 antigen	−0.31 (−0.36, −0.26)	7 × 10^−30^	−0.43 (−0.55, −0.32)	1 × 10^−13^
KIM-1	−0.26 (−0.31, −0.21)	2 × 10^−24^	−0.34 (−0.44, −0.25)	2 × 10^−11^
β2-microglobulin	−0.28 (−0.34, −0.23)	4 × 10^−22^	−0.33 (−0.44, −0.21)	9 × 10^−08^
α1-microglobulin	−0.28 (−0.33, −0.23)	2 × 10^−25^	−0.31 (−0.41, −0.20)	1 × 10^−08^
Cystatin-C	−0.30 (−0.36, −0.24)	1 × 10^−21^	−0.24 (−0.36, −0.13)	6 × 10^−05^
Thrombomodulin	−0.28 (−0.34, −0.23)	3 × 10^−24^	−0.30 (−0.41, −0.20)	3 × 10^−08^
TNFR1	−0.24 (−0.29, −0.19)	5 × 10^−19^	−0.29 (−0.39, −0.18)	2 × 10^−07^
Osteopontin	−0.17 (−0.23, −0.12)	3 × 10^−11^	−0.24 (−0.34, −0.14)	8 × 10^−06^
IL-2 receptor α	−0.22 (−0.27, −0.17)	4 × 10^−17^	−0.18 (−0.28, −0.08)	3 × 10^−04^
Osteoprotegerin	−0.14 (−0.20, −0.09)	8 × 10^−07^	−0.22 (−0.32, −0.12)	3 × 10^−05^
Fibroblast growth factor 21	−0.15 (−0.20, −0.11)	9 × 10^−10^	−0.19 (−0.29, −0.09)	1 × 10^−04^
IGF-binding protein 7	−0.12 (−0.17, −0.07)	8 × 10^−06^	−0.18 (−0.28, −0.08)	5 × 10^−04^
N-terminal prohormone of brain natriuretic peptide	−0.18 (−0.23, −0.12)	3 × 10^−10^	−0.02 (−0.12, 0.08)	7 × 10^−01^
Tissue inhibitor of metalloproteinases 1	−0.13 (−0.18, −0.08)	4 × 10^−07^	−0.18 (−0.27, −0.09)	2 × 10^−04^
Tamm–Horsfall urinary glycoprotein	0.15 (0.09, 0.20)	8 × 10^−08^	0.17 (0.08, 0.27)	6 × 10^−04^
Trefoil factor 3	−0.15 (−0.20, −0.09)	1 × 10^−07^	Not tested	
LC-MS/MS metabolites				
Free sialic acid	−0.28 (−0.34, −0.23)	1 × 10^−21^	−0.32 (−0.44, −0.20)	5 × 10^−07^
SDMA	−0.20 (−0.26, −0.14)	1 × 10^−10^	−0.30 (−0.42, −0.18)	2 × 10^−06^
3-Methyl-histidine	−0.17 (−0.24, −0.11)	7 × 10^−08^	−0.24 (−0.36, −0.13)	3 × 10^−05^
Tryptophan/kynurenine	0.22 (0.17, 0.28)	4 × 10^−15^	0.16 (0.05, 0.27)	4 × 10^−03^
SDMA/ADMA	−0.12 (−0.18, −0.06)	2 × 10^−05^	−0.20 (−0.31, −0.09)	6 × 10^−04^
Free cystine	−0.17 (−0.23, −0.11)	2 × 10^−09^	Not tested	
TMAO	−0.15 (−0.20, −0.09)	1 × 10^−07^	−0.17 (−0.28, −0.07)	1 × 10^−03^
C4DC methylmalonyl/C5OH	−0.15 (−0.21, −0.09)	1 × 10^−06^	−0.16 (−0.27, −0.05)	4 × 10^−03^
Tryptophan	0.16 (0.12, 0.21)	7 × 10^−11^	Not tested	
C5DC (glutaryl) carnitine	−0.13 (−0.18, −0.07)	6 × 10^−06^	−0.14 (−0.25, −0.03)	2× 10^−02^
Methionine	0.14 (0.09, 0.19)	1 × 10^−08^	Not tested	
C4 carnitine	−0.13 (−0.18, −0.08)	3 × 10^−06^	Not tested	
C2 carnitine	−0.12 (−0.17, −0.07)	8 × 10^−06^	Not tested	
C3DC malonyl/3OHB	−0.12 (−0.17, −0.07)	4 × 10^−06^	−0.10 (−0.19, −0.01)	3 × 10^−02^
Citrulline	−0.11 (−0.16, −0.05)	7 × 10^−05^	−0.10 (−0.20, 0.00)	5 × 10^−02^
Threonine	0.11 (0.06, 0.16)	2 × 10^−05^	0.06 (−0.03, 0.15)	2 × 10^−01^
Hydroxyproline	−0.10 (−0.15, −0.05)	9 × 10^−05^	−0.09 (−0.19, 0.00)	6 × 10^−02^
Neopterin	−0.10 (−0.15, −0.05)	9 × 10^−05^	−0.10 (−0.19, −0.00)	5 × 10^−02^
LC-MS/MS tryptic peptides				
Retinal-binding protein 2 (575.8/695.3)	0.09 (0.03, 0.14)	1 × 10^−03^	0.20 (0.11, 0.29)	2 × 10^−05^
Hyaluronan-binding protein 2 (575.2/901.5)	0.04 (−0.02, 0.09)	2 × 10^−01^	0.19 (0.10, 0.28)	5 × 10^−05^
Extracellular glycoprotein lacritin (481.3/501.3)	0.13 (0.08, 0.18)	1 × 10^−07^	0.16 (0.07, 0.25)	9 × 10^−04^
Albumin T70 (501.2/587.5)	0.14 (0.09, 0.19)	1 × 10^−08^	0.04 (−0.06, 0.13)	4 × 10^−01^
Angiotensin II (349.8/136.1)	0.14 (0.09, 0.19)	1 × 10^−07^	0.04 (−0.05, 0.14)	4 × 10^−01^
Cellular repressor of E1A-stimulated genes 1 (575.8/704.4)	0.10 (0.05, 0.15)	4 × 10^−05^	0.14 (0.04, 0.23)	4 × 10^−03^
Chromogranin A (488.2/775.4)	−0.14 (−0.19, −0.09)	2 × 10^−08^	−0.02 (−0.12, 0.07)	6 × 10^−01^
Albumin T6 (575.4/937.4)	0.13 (0.08, 0.18)	2 × 10^−07^	0.12 (0.03, 0.22)	1 × 10^−02^
ApoC-III (598.8/854.4)	−0.12 (−0.17, −0.07)	2 × 10^−06^	−0.07 (−0.17, 0.03)	2 × 10^−01^
Complement C3 (673.4/646.4)	−0.12 (−0.17, −0.07)	4 × 10^−06^	0.04 (−0.05, 0.13)	4 × 10^−01^
Albumin T34 (441.0/680.5)	0.11 (0.06, 0.16)	7 × 10^−06^	0.06 (−0.03, 0.16)	2 × 10^−01^
Haptoglobin (490.5/562.6)	−0.11 (−0.16, −0.06)	2 × 10^−05^	0.01 (−0.09, 0.10)	9 × 10^−01^
Heparin cofactor II (514.8/814.4)	−0.10 (−0.15, −0.05)	6 × 10^−05^	−0.07 (−0.16, 0.03)	2 × 10^−01^
Peroxidase (492.6/703.3)	−0.10 (−0.15, −0.05)	4 × 10^−05^	−0.03 (−0.12, 0.06)	5 × 10^−01^

Regression coefficients are per unit of SD of Gaussianised biomarker

Biomarkers with *p* < 10^-4^ in at least one study are reported, ordered by largest effect size across the two studies

Not tested: the biomarker was not tested for association as it was affected by storage conditions

ADMA, asymmetric dimethylarginine; SDMA, symmetric dimethylarginine; TMAO, trimethylamine-*N*-oxide

When these models were further adjusted for ACR at baseline, the coefficients were reduced somewhat, but the top associations all remained highly significant (ESM Table 3). After adjusting models for the full set of clinical covariates, the largest effect sizes were slightly reduced mainly due to the addition of the weighted average of historical eGFR to the set of covariates, but nine Luminex proteins and four LC-MS/MS measured peptides or metabolites remained significantly associated with final eGFR (ESM Table 4).

Considering the binary outcome of rapid progression, associations were much weaker and fewer were significant (Table 3), but the rank ordering of the associations was similar to that using final eGFR as the outcome.

**Table 3:** Associations of each biomarker (considered separately) with rapid progression from logistic regression models adjusted for age, sex, duration of diabetes, study day eGFR and length of follow-up

Biomarker	SDRNT1BIO		FinnDiane	
	OR (95% CI)	*p* value	OR (95% CI)	*p* value
Luminex proteins				
KIM-1	1.82 (1.52, 2.17)	4 × 10^−11^	2.11 (1.52, 3.00)	2 × 10^−05^
CD27 antigen	1.65 (1.38, 1.99)	9 × 10^−08^	1.89 (1.33, 2.71)	4 × 10^−04^
TNFR1	1.89 (1.54, 2.33)	2 × 10^−09^	1.26 (0.93, 1.73)	1 × 10^−01^
α1-microglobulin	1.86 (1.51, 2.31)	1 × 10^−08^	1.76 (1.28, 2.47)	7 × 10^−04^
Thrombomodulin	1.82 (1.50, 2.22)	2 × 10^−09^	1.72 (1.26, 2.38)	7 × 10^−04^
β2-microglobulin	1.51 (1.25, 1.85)	3 × 10^−05^	1.73 (1.20, 2.56)	4 × 10^−03^
Cystatin-C	1.70 (1.37, 2.14)	3 × 10^−06^	1.44 (1.02, 2.05)	4 × 10^−02^
IL-2 receptor α	1.60 (1.34, 1.91)	2 × 10^−07^	1.27 (0.95, 1.70)	1 × 10^−01^
N-terminal prohormone of brain natriuretic peptide	1.50 (1.24, 1.82)	4 × 10^−05^	1.09 (0.83, 1.43)	5 × 10^−01^
LC-MS/MS metabolites				
Free sialic acid	1.63 (1.33, 2.00)	4 × 10^−06^	1.51 (1.05, 2.20)	3 × 10^−02^
Tryptophan/kynurenine	0.67 (0.55, 0.82)	7 × 10^−05^	0.93 (0.68, 1.26)	6 × 10^−01^
Threonine	0.68 (0.57, 0.80)	8 × 10^−06^	0.80 (0.61, 1.03)	8 × 10^−02^
Methionine	0.69 (0.58, 0.82)	4 × 10^−05^	Not tested	
Tryptophan	0.69 (0.58, 0.82)	4 × 10^−05^	Not tested	

ORs are per unit of SD of Gaussianised biomarker

Biomarkers with *p* < 10^-4^ in at least one study are reported, ordered by largest effect size across the two studies

Not tested: the biomarker was not tested for association as it was affected by storage conditions

### Panels of biomarkers

Table 4 summarises the cross-validated predictive performance of the linear regression models for prediction of achieved eGFR using all the biomarkers with penalty parameters on top of various clinical covariates. On top of age, sex, diabetes duration, baseline eGFR and length of follow-up, the biomarkers from each of the platforms yielded only modest increments in prediction of final eGFR. The Luminex platform gave a similar increment in performance to the LC-MS/MS platform in SDRNT1BIO and somewhat greater performance in FinnDiane. The *r*^2^ increased from 0.47 to 0.58 in SDRNT1BIO, and from 0.33 to 0.48 in FinnDiane when adding the Luminex panel to a model containing age, sex, diabetes duration, eGFR and length of follow-up.

**Table 4:** Cross-validated performance of models for prediction of achieved eGFR

	SDRNT1BIO				FinnDiane			
	Basic covariates		Full covariates		Basic covariates		Full covariates	
Platform	Diff. logLik	*r* ^2^	Diff. logLik	*r* ^2^	Diff. logLik	*r* ^2^	Diff. logLik	*r* ^2^
Baseline	–	0.47	–	0.63	–	0.33	–	0.45
Luminex	86.3	0.58	24.1	0.65	34.5	0.48	14.7	0.50
LC-MS/MS	91.8	0.58	24.7	0.65	23.5	0.43	8.4	0.47
Both platforms	103.2	0.60	29.4	0.65	36.3	0.48	16.5	0.50

Baseline models contain only either basic or full clinical covariates

Basic clinical covariates: age, sex, diabetes duration, study day eGFR, length of follow-up

Full clinical covariates: age, sex, diabetes duration, study day eGFR, length of follow-up, categorical ACR, BMI, DBP, SBP, HbA_1c_, HDL-cholesterol, total cholesterol, smoking status, weighted average of historical eGFR

Differences in test log-likelihood (using natural logarithms) are reported with respect to the baseline model

Diff., difference; logLik, log-likelihood

Similarly, as shown in the AUC data in Table 5, biomarkers from both platforms modestly incremented the prediction of rapid progression of renal disease independently of age, sex, diabetes duration, eGFR and length of follow-up. Both the base model without biomarkers and the model including biomarkers were more predictive in FinnDiane. This is possibly because of longer follow-up and the more advanced stage of renal decline of their participants. Using the metric of expected information for discrimination rather than AUC, the conclusions are similar, i.e. that the biomarkers provide only modest additional predictive information on top of clinical covariates. For example, the addition of both panels to the set of basic covariates provides 0.3 and 0.4 extra bits of information for the prediction of rapid progression in SDRNT1BIO and FinnDiane, respectively. Note that the expected information for discrimination metric shows that the increment in prediction of rapid progression with biomarkers in FinnDiane is similar or slightly greater than in SDRNT1BIO, consistent with the generally stronger univariate associations. See discussion for further consideration of what the expected information for discrimination shows that an AUC does not.

**Table 5:** Cross-validated performance of models for prediction of rapid progression

Platform	SDRNT1BIO						FinnDiane					
	Basic covariates			Full covariates			Basic covariates			Full covariates		
	Diff. logLik	AUC	*Λ*	Diff. logLik	AUC	*Λ*	Diff. logLik	AUC	*Λ*	Diff. logLik	AUC	*Λ*
Baseline	–	0.51	0	–	0.61	0.3	–	0.70	0.6	–	0.78	1.3
Luminex	25.4	0.65	0.3	6.2	0.64	0.4	8.8	0.74	0.9	0.3	0.78	1.4
LC-MS/MS	12.2	0.60	0.2	1.2	0.62	0.3	5.2	0.72	0.9	−0.6	0.78	1.6
Both platforms	22.1	0.63	0.3	3.8	0.63	0.4	7.8	0.73	1	−0.1	0.78	1.8

Baseline models contain only either basic or full clinical covariates

Basic clinical covariates: age, sex, diabetes duration, study day eGFR, length of follow-up

Full clinical covariates: age, sex, diabetes duration, study day eGFR, length of follow-up, categorical ACR, BMI, DBP, SBP, HbA_1c_, HDL-cholesterol, total cholesterol, smoking status, weighted average of historical eGFR

Differences in test log-likelihood (using natural logarithms) are reported with respect to the baseline model

The expected information for discrimination *Λ* is reported in bits

Diff., difference; logLik, log-likelihood

**Fig. 1:**
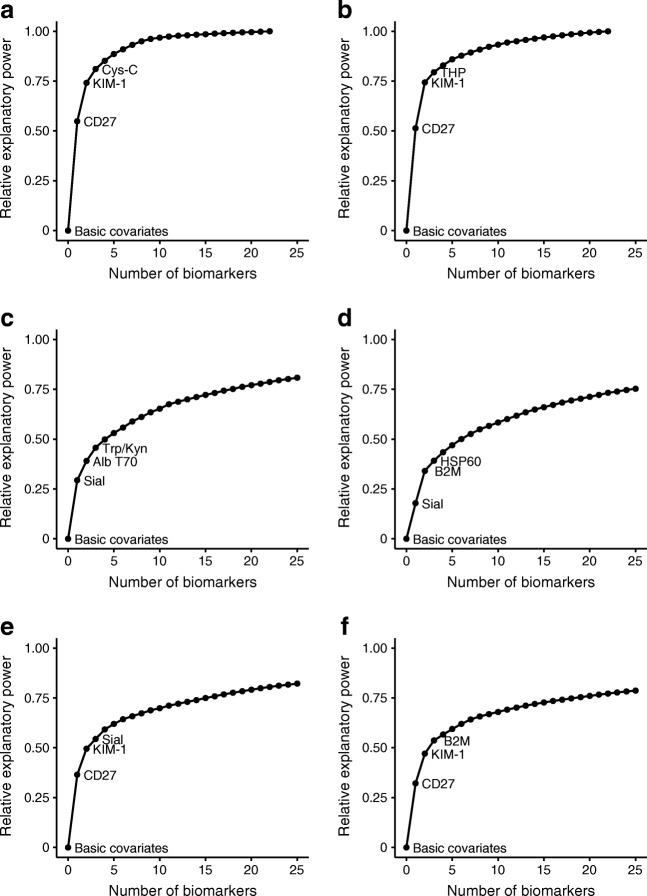
Contribution of biomarker sets to prediction of achieved eGFR when starting from a model containing only age, sex, diabetes duration, study day eGFR and length of follow-up (basic covariates) in SDRNT1BIO (**a**, **c**, **e**) and FinnDiane (**b**, **d**, **f**). The sets of biomarkers added were: (**a**, **b**) Luminex platform; (**c**, **d**) LC-MS/MS platform; (**e**, **f**); both platforms together. Note that for (**c**) to (**f**) the plotting of the curve was interrupted after 25 biomarkers: by definition, the curve would gradually converge to 1 with the addition of all remaining biomarkers. The names of the ten highest ranking biomarkers in each setting are provided in ESM Table 5. Alb T70, albumin T70; B2M, β*-*2-microglobulin, Cys-C, cystatin C, HSP60, heat-shock protein 60 kDa; Sial, free sialic acid; THP, Tamm–Horsfall urinary glycoprotein; Trp/Kyn, tryptophan/kynurenine

By applying the forward selection projection approach, we determined a ranking of the biomarkers within each platform (ESM Table 5). Figure 1 displays the ranking of the first three biomarkers for the two platforms considered separately and jointly, when predicting achieved eGFR in models adjusted for basic clinical covariates. This shows that, in general, the gains in explanatory power flatten out after the first few biomarkers are added to the initial set of clinical covariates. Indeed, for the Luminex platform, 75% of the explanatory power is produced by CD27 and kidney injury molecule 1 (KIM-1) in both cohorts.

**Fig. 2:**
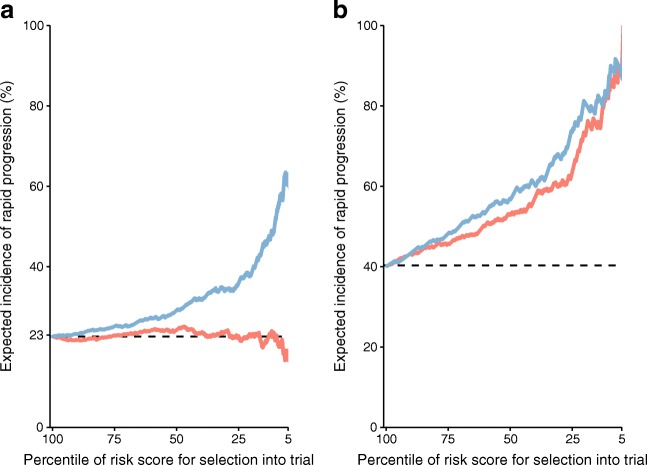
Expected cumulative incidence of rapid progression if a clinical trial was enriched with the top percentile of possible participants according to their risk score in SDRNT1BIO (**a**) and FinnDiane (**b**). The baseline model contained only age, sex, diabetes duration, study day eGFR and length of follow-up (red line) or a model augmented with CD27 and KIM-1 (blue line). The observed event rate is represented by the horizontal dashed line

To consider whether the increment in prediction obtained with these biomarkers would be of practical use, we calculated the enrichment in the rate of rapid progression that would be obtained if the biomarkers were measured in potential entrants to a trial with characteristics similar to the cohort participants. By oversampling into the trial according to varying percentile cut-offs for the risk score from models with and without biomarkers, we could produce the curves of Fig. 2. For example, if the most extreme 25% of participants for biomarkers were oversampled then the expected incidence of rapid progression would go from about 23% to 35% in SDRNT1BIO. By contrast in FinnDiane, where most participants were already albuminuric and rapid progression rates are higher, the enrichment would be less, going from 63% to 73%.

## Discussion

The key findings from this study were that, despite a large number of the biomarkers evaluated showing highly significant associations with eGFR and its decline: (1) the marginal increment in prediction using these biomarkers panels is modest; (2) almost all of the predictive information can be obtained using just two biomarkers (CD27 and KIM-1); and (3) using weighted historical eGFR and albuminuria gives similar prediction performance to using the biomarkers. Nonetheless, in some clinical trial settings where historical records are not available, oversampling those with the highest levels of CD27 and KIM-1 may be useful for stratification into the trial.

Our data emphasise that, while identifying biomarkers that are associated with eGFR and its decline is rather straightforward, building a biomarker panel is not as simple as considering the top few strongly associated biomarkers. The creation of biomarker sets that are jointly predictive needs to take account of the correlation between the biomarkers. When this is done we can see that almost all the gains in prediction are from just a few biomarkers, here CD27 and KIM-1.

In the SUMMIT study (Surrogate Markers for Micro-and Macrovascular Hard Endpoints for Innovative Diabetes Tools), we have previously shown that KIM-1 was the most useful of a wide range of biomarkers associated with eGFR loss in type 2 diabetes [14]. KIM-1 is expressed on the apical membrane of kidney proximal tubule cells. It is well established as a urinary marker of acute kidney injury. Serum KIM-1 has previously been reported to predict eGFR decline and incidence of ESRD in type 1 diabetes [15, 16]. Furthermore, a Mendelian randomisation approach utilising genetic data to infer causality suggested that urinary KIM-1 would be a causal risk factor for decline in eGFR [17]. However, it remains an open question whether it is a causal biomarker or not.

CD27 is a member of the tumour necrosis factor (TNF) receptor superfamily and is required for the maintenance of T cell immunity. We are not aware of previous reports of CD27 in DKD. However, other members of this superfamily (TNF receptor [TNFR]1 and TNFR2) have previously been reported as biomarkers of eGFR decline by us and others in type 2 diabetes [18, 19] and type 1 diabetes [20]. While circulating levels of CD27 correlate with structural renal abnormalities [18] and while there is considerable evidence for a role of the immune system and inflammation in DKD, whether these associations of members of the TNF receptor superfamily with DKD are causal or secondary to altered filtration remains unclear.

A key strength of our study was the deliberate use of two cohorts with differing characteristics and rates of disease. The SDRNT1BIO cohort was not oversampled for albuminuria but the FinnDiane cohort set used was. Accordingly, for similar starting eGFR, the rate of progression in FinnDiane was higher. In general, biomarker associations with final eGFR were stronger among those with albuminuria and the increment in prediction gained with biomarkers was slightly greater in the FinnDiane cohort. The differing storage conditions in the two cohorts (−80°C with no freeze/thaw cycles in SDRNT1BIO and −20°C in FinnDiane) allowed us to examine which of these biomarkers are highly sensitive to storage—an important consideration for their practical deployment in clinical settings.

Another key strength of our study is the use of advanced statistical methods that avoid over-optimistic assessments of prediction. These include cross-validation and use of penalty parameters that account for the high number of analytes being evaluated. We also introduce here a new measure of prediction performance—the expected information for discrimination. See [11] for the full derivation and discussion of this measure, but an important advantage is that it scales linearly so one can directly compare the increment in prediction gained from two differing base levels. As shown in Table 5, the AUC increases from 0.51 to 0.63 with both biomarker platforms in SDRNT1BIO, but from 0.70 to 0.73 in FinnDiane. Although the reader might be misled into thinking the increment in prediction is therefore greater in SDRNT1BIO, in fact the increase in information that is needed to increase an AUC from the higher starting point of 0.70 to 0.73 (0.4 bits) is actually slightly greater than that needed to increase an AUC from 0.51 to 0.63 (0.3 bits). The reason is that, although not commonly appreciated, in fact the AUC does not scale linearly and one cannot compare the absolute increment in AUC from two differing base levels.

Another important strength of our study is our transparency with regard to showing the effect of adjustment for baseline characteristics and for historical eGFR data. As we recently reviewed [3], many reports of biomarker usefulness are overstated because the marginal gain in prediction beyond baseline and historical eGFR are not reported. In both cohorts examined, the contribution of the biomarkers became much less important when a full set of covariates are used in the model, a result that reflects what was observed in earlier high-dimensional biomarker studies [14, 19]. Of course, while in a clinical setting current and historical measurements are available, these may not be readily accessible in other situations, such as in the screening phase of a clinical trial. In the latter case it may be hard to capture more than the basic set of clinical covariates used in our study. Accordingly, we have provided enrichment plots to give a sense of the potential use of biomarkers in such a context. The increments in prediction we have found are such that a sparse panel of KIM-1 and CD27 is unlikely to be of use in clinical decision making. However, an important point to appreciate is that even a small enrichment of event rate in trials can be worthwhile in terms of sample size, power and resulting cost.

A limitation of our study is the relatively short follow-up and low progression rates in SDRNT1BIO. However, we emphasise that this cohort is representative of the current reality of progression rates in those with stage 2 and 3 DKD in type 1 diabetes. Furthermore, the follow-up time is similar to the length of many trials allowing the challenge of demonstrating drug effects, and the need to oversample those with most progression to be clearly understood. The effect of this shorter follow-up time is that the observed slope and rapid progressor status are less stable and subject to more misclassification in SDRNT1BIO, which will make associations tend more towards the null. Accordingly the biomarker outcome associations were less strong in SDRNT1BIO. However, this lesser progression would not introduce false positive associations. Another limitation is that we have evaluated the marginal improvement in prediction with biomarkers on top of baseline eGFR for predicting final eGFR. However eGFR itself is an imperfect measure of actual GFR [21]. Where measurements are noisy, increments in prediction with biomarkers may be more than would occur if GFR were directly measured. However, since in most settings where biomarkers might be used GFR is not directly measured, it is the increment on eGFR that is most relevant. Another limitation is that we were not able to assess incremental prediction on top of the validated Kidney Failure Risk equation as data on calcium and phosphate levels were not available in most participants [22].

## Conclusions

In summary, despite extensive investigation of a wide range of biomarkers, we can demonstrate only modest increments in prediction of future eGFR. The increment is of a magnitude that may be of use in some trial settings but not in clinical decision making. It remains possible, of course, that other biomarkers, or the same biomarkers assayed on other platforms, might achieve greater performance. We hope that the methodological approach we have adopted and illustrated in this work will be useful to others exploring the use of high-dimensional biomarker platforms in a prediction setting.

## Electronic supplementary material

ESM (PDF 619 kb)

## Data Availability

We do not have governance permissions to share individual level data on which these analyses were conducted since they derive from clinical record data. However, for any bona fide requests to audit the validity of the analyses, the verifiable research pipeline which we operate means that one can request to view the analyses being run and the same tabulations resulting. We are also happy to share summary statistics for those wishing to conduct meta-analyses with other studies.

## Abbreviations

ACR: Albumin/creatinine ratio
AUC: Area under the receiver operating characteristic curve
CD27: CD27 antigen
DBP: Diastolic BP
DKD: Diabetic kidney disease
ESRD: End-stage renal disease
FinnDiane: The Finnish Diabetic Nephropathy study
IQR: Interquartile range
KIM-1: Kidney injury molecule 1
LC-MS/MS: LC electrospray tandem MS
RRT: Renal replacement therapy
SBP: Systolic BP
SDRNT1BIO: Scottish Diabetes Research Network Type 1 Bioresource
TNF: Tumour necrosis factor
TNFR: Tumour necrosis factor receptor
