# Supplementary material for: Biomarker panels associated with progression of renal disease in type 1 diabetes
Source: Diabetologia. 2019 Jun 20;62(9):1616–27. doi: 10.1007/s00125-019-4915-0 (PMC6677704; doi:10.1007/s00125-019-4915-0)
Supplement: Supplementary file 1 — (PDF 619 kb) [file 125_2019_4915_MOESM1_ESM.pdf]

Electronic Supplementary Materials Biomarker panels associated with progression of renal disease in type 1 diabetes

Marco Colombo, Erkka Valo, Stuart J. McGurnaghan, Niina Sandholm, Luke A.K. Blackbourn, R. Neil Dalton, David Dunger, Per-Henrik Groop, Paul M. McKeigue, Carol Forsblom, Helen M. Colhoun on behalf of the FinnDiane Study Group and the Scottish Diabetes Research Network (SDRN) Type 1 Bioresource Collaboration

ESM Methods

Use of hierarchical shrinkage priors

We adopted a Bayesian modelling approach based on hierarchical shrinkage priors [1], in which the clinical covariates used to control for confounding in the models were assigned a Gaussian prior (which induces some shrinkage), while the biomarkers were penalised through the horseshoe prior to promote sparsity [2], [3]. Standard methods for penalized regression include ridge regression (equivalent to a Gaussian prior on all coefficients) or LASSO regression (equivalent to a double exponential prior). Hierarchical shrinkage priors (such as the horseshoe) are more flexible than either of these, allowing the model for instance to learn a spike-and-slab distribution of effect sizes (in which a few variables have large effects but most have near zero effects) if that is what the data support. Developments in Bayesian computation, implemented in programs such as Stan and PyMC3, have made it possible to use these more flexible priors, even when some variables have high posterior correlations.

Note on the expected information for discrimination

For logistic regression models besides reporting the area under the ROC curve (AUC), we also presented the expected information for discrimination Λ expressed in bits [4]. The expected information for discrimination is the average weight of evidence (log-likelihood ratio) favouring true (case or control) over false (control or case) status, where the average is taken over the sampling distributions of the weight of evidence in cases and controls. A key advantage of using the information for discrimination is that the contributions of independent predictors are additive. Therefore, this is a better measure of the incremental contribution of biomarkers to the predictive performance, as it captures the amount of additional information that they contain over and beyond the initial set of clinical covariates. Computations were done with the R package `wevid` (version 0.6: https://CRAN.R-project.org/package=wevid).

Note on the projection predictive method

To recover a sparse model, we applied a projection approach according to which the high-dimensional posterior draws of the model containing all biomarkers (full model) are projected to lower-dimensional subspaces [5], [6]. The advantages of the projection predictive method for variable selection have been discussed by Piironen and Vehtari [7]. The method compares the predictive distribution of each candidate model based on a subset of variables to the predictive distribution of the full model, using the Kullback-Leibler (KL) divergence to evaluate the discrepancy between the candidate model and the full model. By fitting the candidate model to the predictive distribution rather than to the target variable, overfitting is reduced.

ESM References

[1] Peltola T, Havulinna AS, Salomaa V, Vehtari A. Hierarchical Bayesian survival analysis and projective covariate selection in cardiovascular event risk prediction. In: Laskey KB, Jones J, Almond R, editors. Proceedings of eleventh UAI Bayesian Modeling Applications Workshop (BMAW 2014), CEUR workshop proceedings, vol. 1218, 2014, pp. 79–88.

[2] Carvalho C, Polson N, Scott J. The horseshoe estimator for sparse signals. Biometrika 2010;97:465–80.

[3] Piironen J, Vehtari A. On the hyperprior choice for the global shrinkage parameter in the horseshoe prior. In:. Proceedings of the 20th international conference on artificial intelligence and statistics (AISTATS), PMLR, vol. 54, 2017, pp. 905–13.

[4] McKeigue P. Quantifying performance of a diagnostic test as the expected information for discrimination: Relation to the C-statistic. Statistical Methods in Medical Research 2018.

[5] Goutis C, Robert CP. Model choice in generalised linear models: A Bayesian approach via Kullback-Leibler projections. Biometrika 1998;85:29–37.

[6] Dupuis J, Robert CP. Variable selection in qualitative models via an entropic explanatory power. Journal of Statistical Planning and Inference 2003;111:77–94.

[7] Piironen J, Vehtari A. Comparison of Bayesian predictive methods for model selection. Statistics and Computing 2017;27:711–35.

ESM Table 1: Participant characteristics at study day stratified by rapid progression status

	SDRNT1BIO		FinnDiane	
Covariate	Stable (N = 665)	Rapid progressor (N = 194)	Stable (N = 188)	Rapid progressor (N = 127)
**Main characteristics**				
Age (years)	55.7 (46.2, 64.5)	54.5 (45.9, 63.8)	46.8 (37.8, 53.7)	43.6 (33.8, 50.7)
Sex (Female)	58.2%	52.1%	47.9%	40.2%
Diabetes duration (years)	26.4 (16.9, 37.9)	26.7 (18.9, 36.5)	33.4 (26.9, 39.1)	29.9 (24.3, 34.8)
**Kidney function**				
eGFR (ml/min/1.73m²)	72.5 (62.8, 83.7)	70.2 (57.3, 85.4)	61.1 (47.5, 68.4)	54.3 (43.6, 64.0)
Achieved eGFR (ml/min/1.73m²)	76.6 (64.2, 88.3)	53.3 (32.8, 74.1)	51.8 (30.9, 64.3)	10.0 (10.0, 10.0)
Weighted average of historical eGFR (ml/min/1.73m²)	81.5 (70.6, 91.3)	80.2 (70.7, 91.9)	68.2 (58.6, 78.0)	58.7 (45.8, 71.9)
Albumin/creatinine ratio (mg/mmol)	0.5 (0.3, 1.2)	1.1 (0.4, 10.2)	11.8 (4.7, 43.3)	56.3 (20.5, 129.5)
ACR category (Normo/Micro/Macro)	87.9%/10.0%/2.1%	67.7%/13.4%/18.8%	0%/37.8%/62.2%	0%/11.8%/88.2%
Prospective eGFR slope (ml/min/1.73m²/year)	-0.1 (-1.4, 1.1)	-5.2 (-6.7, -4.0)	-1.3 (-2.1, -0.3)	-5.0 (-7.1, -3.9)
**Other covariates**				
Length of follow-up (years)	5.2 (4.5, 5.7)	5.1 (4.4, 5.7)	10.3 (7.2, 13.3)	6.5 (4.8, 9.8)
HbA1c (mmol/mol)	67 (60, 77)	73 (64, 88)	68 (58, 76)	79 (69, 88)
HbA1c (%)	8.3 (7.6, 9.2)	8.8 (8.0, 10.2)	8.4 (7.5, 9.1)	9.4 (8.5, 10.2)
Body mass index (kg/m²)	27.1 (24.6, 30.3)	27.4 (24.6, 30.7)	26.0 (23.2, 28.0)	25.6 (23.4, 28.7)
HDL-C (mmol/l)	1.6 (1.3, 1.9)	1.5 (1.2, 1.9)	0.9 (0.7, 1.1)	0.8 (0.6, 1.0)
Total cholesterol (mmol/l)	4.5 (3.9, 5.1)	4.5 (4.0, 5.3)	5.0 (4.4, 5.6)	5.3 (4.7, 6.0)
Systolic blood pressure (mmHg)	133 (121, 144)	137 (126, 151)	141 (130, 154)	150 (132, 159)
Diastolic blood pressure (mmHg)	74 (68, 80)	75 (68, 82)	80 (71, 87)	84 (78, 90)
Ever smoker	64.2%	65.5%	47.9%	58.3%
On any anti-hypertensive treatment	59.2%	70.1%	93.6%	95.3%
On ACE or ARB	53.8%	65.5%	85.6%	86.6%

We report median and interquartile range (IQR) for continuous variables, and frequency for categorical variables
Stable: prospective eGFR slope ≥ -3ml min^{-1}[1.73m]$^{-2}$
Rapid progressor: prospective eGFR slope < -3ml min^{-1}[1.73m]$^{-2}$

ESM Table 2: Summary characteristics of all biomarkers measured in the study

		SDRNT1BIO			FinnDiane		
Biomarker	Units	Median (IQR)	Range	Below	Median (IQR)	Range	Below
**Luminex proteins**							
Alpha-1-Microglobulin	µg/mL	17.0 (14.0, 22.0)	4.3 – 77.5		31.0 (23.0, 40.0)	6.6 – 119.0	
Beta-2-Microglobulin	µg/mL	2.3 (1.8, 2.9)	0.9 – 8.3		3.9 (3.0, 5.2)	1.0 – 13.0	
Calbindin	ng/mL	Intraclass correlation < 0.4		51.2	Affected by storage conditions		43.1
CD27 antigen	U/mL	42.0 (29.0, 69.0)	0.4 – 510.0	0.1	183.8 (112.0, 301.5)	19.0 – 867.0	
Clusterin	µg/mL	Intraclass correlation < 0.4			Affected by storage conditions		
Cystatin-C	ng/mL	758.0 (632.5, 948.0)	333.0 – 2100.0		1110.0 (900.5, 1370.0)	365.0 – 4480.0	
Eotaxin-2	pg/mL	884.0 (569.5, 1420.0)	51.0 – 6510.0		Affected by storage conditions		
Fatty Acid-Binding Protein, heart	ng/mL	4.6 (4.6, 4.6)	4.6 – 77.0	90.6	Intraclass correlation < 0.4		84.8
Fibroblast Growth Factor 21	ng/mL	0.1 (0.1, 0.3)	0.1 – 2.8	38.3	0.3 (0.2, 0.5)	0.1 – 5.3	
Fibroblast Growth Factor 23	ng/mL	0.1 (0.1, 0.1)	0.0 – 2.7	88.0	0.1 (0.0, 0.1)	0.0 – 0.8	30.2
Growth-Regulated alpha protein	pg/mL	123.0 (92.0, 173.0)	13.0 – 968.0		128.0 (90.5, 189.0)	21.0 – 653.0	
Insulin-like Growth Factor-Binding Protein 7	ng/mL	43.0 (36.0, 49.0)	9.3 – 90.0		53.0 (45.0, 64.0)	6.6 – 166.5	
Interleukin-1 receptor type 1	pg/mL	1250.0 (1060.0, 1460.0)	216.0 – 2840.0		Affected by storage conditions		
Interleukin-1 receptor type 2	ng/mL	8.6 (7.2, 10.0)	1.6 – 19.0		Affected by storage conditions		
Interleukin-2 receptor alpha	pg/mL	2620.0 (2100.0, 3340.0)	955.0 – 12600.0		3250.0 (2475.0, 4165.0)	1130.0 – 12100.0	
Kidney Injury Molecule-1	ng/mL	0.0 (0.0, 0.1)	0.0 – 1.3	47.3	0.1 (0.0, 0.2)	0.0 – 2.5	16.1
Latency-Associated-Peptide	ng/mL	11.0 (8.6, 12.8)	0.6 – 20.0		9.2 (6.8, 12.0)	0.8 – 23.0	
Lectin-Like Oxidized LDL Receptor 1	ng/mL	0.8 (0.3, 1.3)	0.3 – 7.1	29.3	0.3 (0.3, 1.1)	0.3 – 6.6	53.4
Neutrophil Gelatinase-Associated Lipocalin	ng/mL	381.0 (282.0, 519.0)	58.0 – 1410.0		Affected by storage conditions		
N-terminal prohormone of brain natriuretic peptide	pg/mL	289.0 (136.0, 632.5)	7.5 – 10650.0	0.6	39.0 (7.5, 182.5)	7.5 – 7690.0	35.5
Osteopontin	ng/mL	12.0 (8.5, 17.0)	0.9 – 65.0	0.1	9.1 (6.4, 13.0)	0.9 – 54.0	0.6
Osteoprotegerin	pM	6.8 (5.5, 8.4)	0.6 – 19.0		6.6 (5.4, 8.1)	1.8 – 16.0	
Progranulin	ng/mL	11.0 (7.9, 16.0)	2.4 – 2190.0	6.4	14.0 (11.0, 21.0)	2.4 – 276.0	2.1
Tamm-Horsfall Urinary Glycoprotein	µg/mL	0.1 (0.0, 0.1)	0.0 – 0.1	0.1	0.1 (0.0, 0.1)	0.0 – 0.1	
Thrombomodulin	ng/mL	5.0 (4.2, 6.2)	1.8 – 19.0		7.7 (5.7, 10.0)	2.3 – 22.0	
Thymus-Expressed Chemokine	pg/mL	275.0 (154.0, 457.0)	39.0 – 4040.0	4.0	277.0 (180.5, 437.0)	47.5 – 2670.0	6.7
Tissue Inhibitor of Metalloproteinases 1	ng/mL	141.0 (123.0, 163.0)	65.0 – 340.0		168.0 (142.0, 193.0)	70.0 – 323.0	
Trefoil Factor 3	µg/mL	0.1 (0.1, 0.2)	0.0 – 3.0		Affected by storage conditions		
Tumor Necrosis Factor Receptor I	pg/mL	1760.0 (1340.0, 2385.0)	99.0 – 8340.0		2700.0 (1905.0, 3620.0)	242.0 – 11300.0	
Vascular Endothelial Growth Factor	pg/mL	256.0 (167.0, 388.0)	21.0 – 1060.0	0.4	276.0 (155.0, 446.0)	21.0 – 2030.0	2.3
**LC-MSMS metabolites**							
1-Methyl-histidine	µM/L	5.1 (2.2, 11.8)	0.2 – 79.0	3.6	5.6 (2.9, 12.5)	0.2 – 150.0	0.6
3-hydroxykynurenine	nM/L	Excessive left-censoring		99.9	Excessive left-censoring		98.2
3-hydroxytyramine HCl [conf]	µM/L	0.1 (0.1, 0.1)	0.1 – 9.6	97.7	Excessive left-censoring		98.5
3-hydroxytyramine HCl	µM/L	0.1 (0.1, 0.1)	0.1 – 7.0	97.7	Excessive left-censoring		98.2
3-Methyl-histidine	µM/L	4.5 (3.4, 6.0)	0.7 – 24.2		7.1 (5.1, 9.8)	1.0 – 33.8	
3OMD	nM/L	189.0 (161.0, 219.0)	69.0 – 3788.0		Intraclass correlation < 0.4		
5-HIAA	nM/L	Excessive left-censoring		99.7	Excessive left-censoring		98.2
5-hydroxytryptophan	nM/L	Excessive left-censoring		99.8	Excessive left-censoring		98.5
8OHdG	nM/L	Excessive left-censoring		99.7	Excessive left-censoring		97.9
Adenine [conf]	nM/L	Excessive missingness at random			Excessive missingness at random		
Adenine	nM/L	Excessive missingness at random			Excessive missingness at random		
Adenosine	nM/L	Excessive left-censoring		99.1	Excessive left-censoring		98.2
ADMA	nM/L	468.5 (411.6, 523.0)	270.0 – 958.0		478.0 (431.0, 537.0)	220.0 – 769.0	
Alanine	µM/L	452.5 (383.9, 525.9)	152.0 – 1329.3		Affected by storage conditions		
Anthranilic acid	nM/L	Excessive left-censoring		99.9	250.0 (250.0, 250.0)	250.0 – 1043.0	91.2
Arginine	µM/L	116.9 (102.1, 134.9)	52.0 – 341.3		Affected by storage conditions		
Argininosuccinic acid	µM/L	Excessive left-censoring		99.9	Excessive left-censoring		98.5
Argininosuccinic anhydride	µM/L	Excessive left-censoring		99.9	Excessive left-censoring		98.5
Aspartic acid	µM/L	24.5 (20.4, 29.6)	11.1 – 49.9		Affected by storage conditions		

ESM Table 2: Summary characteristics of all biomarkers measured in the study *(continued)*

Biomarker	Units	SDRNT1BIO			FinnDiane		
		Median (IQR)	Range	Below	Median (IQR)	Range	Below
Beta-Alanine	µM/L	9.8 (8.4, 11.8)	4.3 – 83.9		9.5 (8.3, 11.3)	5.2 – 46.5	
C10:1 Carnitine	nM/L	99.0 (69.0, 141.0)	5.0 – 525.0	0.6	80.5 (43.0, 125.0)	5.0 – 403.0	1.8
C10 Carnitine	nM/L	157.0 (100.0, 252.1)	15.0 – 1472.0	1.5	149.5 (88.5, 252.8)	15.0 – 1490.0	2.1
C12 Carnitine	nM/L	5.0 (5.0, 10.0)	5.0 – 58.5	73.7	8.0 (2.5, 17.8)	2.5 – 81.0	32.3
C14:1 Carnitine	nM/L	399.5 (320.0, 519.0)	60.0 – 1443.0	0.3	410.5 (308.2, 557.5)	60.0 – 3433.0	9.1
C14 Carnitine	nM/L	125.0 (92.2, 163.0)	25.0 – 461.0	1.6	122.0 (98.0, 161.0)	25.0 – 489.0	1.8
C16:1OH Carnitine	nM/L	20.0 (10.0, 29.0)	10.0 – 100.0	48.1	10.0 (10.0, 26.0)	10.0 – 63.0	52.2
C16 Carnitine	nM/L	309.0 (245.2, 396.0)	82.0 – 1027.0		271.0 (202.0, 350.8)	117.0 – 814.0	
C16OH Carnitine	nM/L	10.0 (10.0, 10.0)	10.0 – 65.0	89.9	10.0 (10.0, 10.0)	10.0 – 43.0	93.3
C18 Carnitine	nM/L	157.0 (116.0, 206.0)	25.0 – 514.0	2.8	140.0 (92.8, 200.0)	25.0 – 1354.0	6.2
C18OH Carnitine	nM/L	10.0 (10.0, 10.0)	10.0 – 100.0	82.1	Intraclass correlation < 0.4		85.6
C2 Carnitine	µM/L	7.0 (5.4, 9.3)	1.9 – 23.9		Affected by storage conditions		93.0
C3 Carnitine	nM/L	421.5 (323.0, 551.0)	101.0 – 5230.0		133.0 (85.1, 187.0)	25.0 – 798.0	7.9
C3DC Malonyl/3OHB	nM/L	137.0 (99.0, 216.8)	25.0 – 2927.0	1.4	131.0 (89.0, 206.8)	25.0 – 1416.0	2.9
C4 Carnitine	nM/L	212.0 (155.2, 282.0)	53.0 – 1394.0	0.1	Affected by storage conditions		2.9
C4DC Methylmalonyl/C5OH	nM/L	125.0 (100.0, 158.0)	50.0 – 707.0		166.5 (132.2, 222.0)	64.0 – 662.0	
C4DC Methylmalonyl	nM/L	Intraclass correlation < 0.4		66.9	13.0 (10.0, 26.0)	10.0 – 85.0	48.7
C5 Carnitine	nM/L	114.0 (89.0, 146.8)	25.0 – 1000.0	1.6	109.2 (83.2, 147.0)	25.0 – 10305.0	3.2
C5DC (Glutaryl) Carnitine [conf]	nM/L	105.0 (86.0, 133.0)	25.0 – 413.0	1.8	124.0 (95.2, 163.5)	25.0 – 482.0	1.2
C5DC (Glutaryl) Carnitine	nM/L	135.0 (109.0, 165.8)	25.0 – 489.0	0.2	163.5 (127.0, 230.4)	25.0 – 718.0	0.3
C5OH specific	nM/L	25.0 (25.0, 25.0)	25.0 – 140.0	76.1	25.0 (25.0, 58.0)	25.0 – 157.0	68.0
C6 Carnitine	nM/L	52.0 (36.0, 74.0)	11.0 – 276.0		22.0 (14.0, 37.0)	5.0 – 113.0	12.0
C6DC (methylglutaryl) Carnitine	nM/L	82.0 (58.0, 112.0)	25.0 – 662.0	17.2	92.0 (62.0, 135.0)	25.0 – 684.0	15.0
C8 Carnitine	nM/L	108.0 (70.0, 158.8)	5.0 – 958.0	0.1	115.0 (79.0, 177.8)	5.0 – 673.0	0.3
Carnosine	nM/L	Intraclass correlation < 0.4		83.5	Affected by storage conditions		1.2
Citrate [conf]	µM/L	Excessive missingness at random			Excessive missingness at random		1.8
Citrate	µM/L	Excessive missingness at random			Excessive missingness at random		0.3
Citrulline	µM/L	41.0 (34.1, 50.1)	8.3 – 108.3		43.8 (36.8, 51.6)	14.5 – 128.0	
Creatine	µM/L	20.5 (14.0, 32.3)	4.4 – 576.0		19.6 (14.1, 28.2)	7.1 – 110.7	
Cystathionine [conf]	nM/L	130.0 (85.0, 202.8)	12.5 – 3589.0	9.2	Affected by storage conditions		32.6
Cystathionine	nM/L	134.0 (88.0, 204.0)	7.5 – 3682.0	3.9	Affected by storage conditions		18.8
Dihydrothymine	µM/L	Excessive left-censoring		99.9	Excessive left-censoring		98.2
Dihydrouracil	µM/L	Excessive left-censoring		99.9	Excessive left-censoring		98.2
Free Carnitine	µM/L	34.2 (28.9, 40.8)	9.3 – 77.1		Affected by storage conditions		
Free Cystine	µM/L	41.4 (33.3, 51.5)	12.9 – 113.6		Affected by storage conditions		14.4
Free homocystine [conf]	µM/L	Excessive left-censoring		99.0	Excessive left-censoring		97.4
Free homocystine	µM/L	Excessive left-censoring		99.1	Excessive left-censoring		98.2
Free Sialic acid	nM/L	793.0 (650.5, 1014.0)	325.0 – 6369.0		983.0 (789.2, 1300.8)	336.0 – 3362.0	
Galactose-1-Phosphate [conf]	µM/L	Excessive missingness at random			Excessive missingness at random		
Galactose-1-Phosphate	µM/L	0.8 (0.4, 1.4)	0.1 – 9.4	11.1	0.7 (0.1, 1.6)	0.1 – 15.6	34.3
Glutamate	µM/L	61.6 (51.0, 73.2)	25.3 – 192.5		Affected by storage conditions		
Glutamate/Glutamine	ratio	0.1 (0.1, 0.1)	0.1 – 0.4		Affected by storage conditions		
Glutamine	µM/L	562.4 (503.9, 631.0)	284.2 – 1239.0		Affected by storage conditions		3.5
Glycine	µM/L	281.4 (237.5, 342.1)	128.0 – 791.3		312.1 (260.8, 365.5)	157.7 – 802.2	
Glycine/Valine	ratio	1.2 (0.9, 1.6)	0.3 – 7.7		1.3 (1.1, 1.7)	0.5 – 5.5	
Guanidinoacetic acid	µM/L	2.4 (1.9, 2.9)	0.8 – 7.2		2.3 (1.8, 2.7)	0.8 – 6.0	
Hexitol	µM/L	10.4 (7.4, 16.2)	3.4 – 599.2		13.8 (9.0, 21.8)	1.1 – 228.9	0.3
Histidine	µM/L	84.9 (77.0, 95.5)	37.7 – 341.8		81.3 (70.5, 91.1)	38.0 – 142.0	
HMMA	nM/L	Excessive left-censoring		99.9	Excessive left-censoring		97.9
Homovanillic acid	nM/L	718.0 (476.0, 1032.5)	125.0 – 416201.0	4.5	1057.0 (643.5, 1562.0)	125.0 – 4552.0	1.2
Hydroxyproline	µM/L	12.2 (8.9, 16.8)	3.4 – 69.8		17.6 (13.3, 25.7)	3.5 – 64.9	
Hypoxanthine	µM/L	7.8 (6.1, 10.2)	0.5 – 45.2	0.2	7.8 (5.5, 10.2)	0.5 – 24.0	0.6

ESM Table 2: Summary characteristics of all biomarkers measured in the study *(continued)*

		SDRNT1BIO			FinnDiane		
Biomarker	Units	Median (IQR)	Range	Below	Median (IQR)	Range	Below
Inosine	nM/L	1137.0 (530.2, 2711.1)	25.0 – 15287.0	0.3	1339.0 (587.0, 3068.0)	25.0 – 15287.0	3.5
Isoleucine	µM/L	72.2 (57.9, 90.5)	21.1 – 247.6		84.8 (70.5, 100.7)	32.8 – 177.9	
Kynurenic acid	nM/L	61.0 (48.0, 78.0)	10.0 – 262.0	0.4	82.5 (62.0, 109.0)	28.0 – 404.0	
Kynurenine	µM/L	2.9 (2.4, 3.6)	1.3 – 8.7		3.0 (2.4, 3.7)	1.0 – 9.5	
Leucine/Alanine	ratio	0.3 (0.2, 0.3)	0.1 – 1.1		0.3 (0.3, 0.4)	0.2 – 0.7	
Leucine	µM/L	125.5 (102.0, 154.6)	34.1 – 426.4		151.8 (126.4, 179.9)	61.3 – 306.9	
Lysine	µM/L	197.3 (170.4, 223.0)	88.6 – 474.3		201.3 (180.9, 230.8)	99.3 – 368.7	
Malate	nM/L	Intraclass correlation < 0.4		55.1	134.8 (50.0, 202.5)	50.0 – 1025.0	32.3
Methionine	µM/L	26.5 (22.2, 31.5)	5.8 – 71.0		Affected by storage conditions		0.3
Methylcitrate	nM/L	1211.5 (723.2, 2010.5)	125.0 – 23028.0	5.0	Affected by storage conditions		2.3
Methymalonic acid	nM/L	304.0 (234.0, 415.0)	25.0 – 76024.0	0.2	291.5 (225.0, 373.0)	25.0 – 1465.0	0.6
N-acetylaspartate [conf]	nM/L	1045.5 (869.5, 1238.0)	432.0 – 1947.0		620.5 (524.0, 757.0)	190.0 – 1554.0	
N-acetylaspartate	nM/L	679.0 (574.0, 808.8)	310.0 – 1757.0		576.0 (485.0, 750.0)	125.0 – 1554.0	0.9
N-acetylglutamine [conf]	nM/L	Excessive missingness at random			446.5 (175.0, 583.2)	175.0 – 1146.0	29.0
N-acetylglutamine	nM/L	392.5 (175.0, 516.5)	175.0 – 978.0	43.5	Excessive missingness at random		
NAG	µmol/L/min	30.0 (25.3, 35.5)	12.3 – 71.2		25.0 (21.2, 30.9)	11.0 – 64.8	
Neopterin	nM/L	7.5 (7.5, 7.5)	7.5 – 32.0	91.2	7.5 (7.5, 7.5)	7.5 – 53.5	76.0
Nitrotyrosine	nM/L	Excessive left-censoring		99.9	Excessive left-censoring		98.2
Ornithine	µM/L	100.8 (86.6, 121.8)	44.4 – 272.3		91.5 (79.8, 107.0)	36.1 – 183.4	
Orotic	nM/L	Intraclass correlation < 0.4			Intraclass correlation < 0.4		
Phenylalanine	µM/L	75.8 (67.7, 85.5)	34.1 – 222.5		82.4 (71.5, 93.0)	34.4 – 144.8	
Phenylalanine/Tyrosine	ratio	1.0 (0.9, 1.2)	0.5 – 3.2		1.2 (1.0, 1.4)	0.6 – 2.8	
Phosphoethanolamine	µM/L	Excessive left-censoring		99.9	Excessive left-censoring		98.5
Phosphoserine [conf]	µM/L	Excessive missingness at random			Excessive missingness at random		
Phosphoserine	µM/L	7.3 (5.5, 11.1)	1.3 – 25.8		Intraclass correlation < 0.4		0.6
Proline	µM/L	212.9 (173.9, 257.7)	79.5 – 778.5		Affected by storage conditions		
Pyridoxal 5prime-phosphate [conf]	nM/L	Excessive left-censoring		99.9	Excessive left-censoring		98.2
Pyridoxal 5prime-phosphate	nM/L	Excessive left-censoring		99.9	Excessive left-censoring		98.2
Pyroglutamic acid	µM/L	25.0 (25.0, 25.0)	25.0 – 98.5	97.4	Affected by storage conditions		
Quinolinic acid	nM/L	Intraclass correlation < 0.4			1437.0 (1107.2, 1959.5)	410.0 – 10322.0	
Sarcosine	µM/L	6.1 (2.5, 8.4)	2.5 – 69.5	42.1	5.7 (2.5, 7.7)	2.5 – 46.1	38.4
SDMA/ADMA	ratio	1.2 (1.1, 1.4)	0.7 – 3.7		1.4 (1.2, 1.7)	0.8 – 3.7	
SDMA	nM/L	546.0 (478.0, 651.8)	293.0 – 1881.0		686.0 (563.0, 842.0)	356.0 – 1766.0	
Serine	µM/L	142.1 (123.5, 165.0)	79.0 – 290.3		181.0 (152.0, 201.0)	81.0 – 359.0	
Succinate	µM/L	17.4 (14.6, 20.8)	5.6 – 63.0		Intraclass correlation < 0.4		0.6
Succinylacetone	nM/L	Excessive left-censoring		99.9	Excessive left-censoring		98.5
Sulphocysteine	nM/L	716.5 (590.0, 868.0)	100.0 – 2539.0	0.3	Affected by storage conditions		51.9
Taurine	µM/L	111.7 (86.0, 143.1)	32.4 – 511.1		76.9 (54.5, 101.0)	26.7 – 215.8	
Threonine	µM/L	118.4 (95.7, 140.5)	30.2 – 562.8		113.1 (97.8, 134.5)	45.5 – 218.4	
Thymine	nM/L	Excessive missingness at random		36.4	115.0 (50.0, 139.0)	50.0 – 272.0	20.8
TMAO	µM/L	5.0 (3.0, 8.1)	0.3 – 67.4		6.7 (4.5, 10.1)	1.7 – 156.8	
Tryptophan	µM/L	58.9 (50.8, 67.4)	28.4 – 137.2		Affected by storage conditions		
Tryptophan/Kynurenine	ratio	20.4 (16.2, 24.6)	5.3 – 48.1		18.5 (14.9, 23.7)	4.7 – 52.6	
Tyrosine	µM/L	75.1 (63.1, 90.5)	30.0 – 204.8		69.0 (57.5, 81.2)	37.6 – 137.3	
Uracil	nM/L	970.0 (768.2, 1206.0)	264.0 – 2677.0		Intraclass correlation < 0.4		1.2
Ureidopropionate	nM/L	Excessive left-censoring		99.9	Excessive left-censoring		98.5
Uric acid	µM/L	363.0 (295.0, 459.0)	79.0 – 858.0		231.0 (188.0, 279.0)	15.0 – 601.0	
Valine	µM/L	232.1 (197.7, 268.6)	69.5 – 596.5		232.8 (201.0, 272.2)	101.9 – 436.6	
Xanthine	µM/L	2.0 (1.7, 2.5)	0.2 – 14.2	0.2	1.9 (1.5, 2.4)	0.2 – 12.6	0.3
Xanthurenic acid	nM/L	25.0 (25.0, 119.0)	25.0 – 579.0	50.9	77.0 (25.0, 128.4)	25.0 – 353.0	28.7
**LC-MSMS tryptic peptides**							
ACTIN (758.9 / 765.4)	*	Intraclass correlation < 0.4		0.8	Intraclass correlation < 0.4		

ESM Table 2: Summary characteristics of all biomarkers measured in the study *(continued)*

Biomarker	Units	SDRNT1BIO Median (IQR)	SDRNT1BIO Range	SDRNT1BIO Below	FinnDiane Median (IQR)	FinnDiane Range	FinnDiane Below
Acyloxyacyl hydrolase (703.9 / 931.3)	*	193.8 (151.5, 245.2)	5.0 – 633.6	0.5	194.6 (123.7, 269.7)	19.2 – 735.6	0.3
Afamin (416.8 / 572.4)	*	Intraclass correlation < 0.4		18.2	Intraclass correlation < 0.4		38.4
Afamin (563.8 / 825.4)	*	Intraclass correlation < 0.4		17.6	Intraclass correlation < 0.4		23.8
AGR2 (lung cancer biomarker) (407.7 / 701.4)	*	Intraclass correlation < 0.4		1.0	Intraclass correlation < 0.4		
Albumin T31 (337.3 / 416.3)	*	Intraclass correlation < 0.4		0.4	1897.4 (1677.8, 2174.7)	417.0 – 3948.7	
Albumin T34 (441.0 / 680.5)	*	1192.0 (1051.5, 1353.5)	5.0 – 2520.9	0.4	1016.5 (898.7, 1149.8)	290.9 – 1860.2	
Albumin T6 (575.4 / 937.4)	*	27296.9 (24671.0, 30189.0)	5.0 – 47676.5	0.4	23164.3 (20356.6, 26048.2)	9596.2 – 47832.8	
Albumin T70 (501.2 / 587.5)	*	4303.3 (3687.7, 4970.2)	5.0 – 10234.8	0.4	3181.2 (2453.8, 3845.9)	344.5 – 7405.8	
Alpha-1-acid-glycoprotein 1 (723.3 / 937.4)	*	106.4 (85.9, 131.9)	5.0 – 276.8	0.4	90.2 (75.5, 115.7)	29.0 – 234.3	
Alpha-1-antichymotrypsin iso1 (608.4 / 775.4)	*	236.7 (192.7, 286.9)	5.0 – 980.6	0.4	204.4 (172.4, 241.5)	83.5 – 981.2	
Alpha-1-antitrypsin (444.8 / 718.4)	*	78.0 (61.9, 100.0)	5.0 – 205.0	0.4	74.5 (58.2, 92.3)	18.4 – 201.1	
Alpha-1-antitrypsin (556.4 / 797.7)	*	45.2 (34.2, 58.3)	5.0 – 205.5	0.9	39.9 (30.0, 53.3)	5.0 – 114.9	0.3
Alpha-1-antitrypsin (631.3 / 889.5)	*	62.5 (44.5, 84.1)	5.0 – 193.1	7.1	47.2 (29.9, 63.8)	5.0 – 129.2	12.9
Alpha-1B-glycoprotein (619.3 / 894.5)	*	84.1 (68.2, 104.0)	5.0 – 197.2	0.4	78.3 (62.4, 95.5)	5.0 – 173.0	0.3
Alpha-1B-glycoprotein (687.4 / 960.6)	*	Intraclass correlation < 0.4		4.4	Intraclass correlation < 0.4		2.9
Alpha-2-macroglobulin (697.8 / 737.4)	*	131.5 (99.2, 164.4)	5.0 – 350.6	0.4	164.5 (127.3, 211.5)	54.9 – 460.3	
Alpha-fetoprotein (490.7 / 833.3)	*	47.0 (37.6, 57.2)	5.0 – 120.6	2.6	36.4 (28.8, 44.6)	5.0 – 95.2	3.5
Aminopeptidase B (547.8 / 680.4)	*	Intraclass correlation < 0.4		0.4	Intraclass correlation < 0.4		0.3
Angiotensin II (349.8 / 136.1)	*	1104.0 (967.5, 1369.5)	5.0 – 2393.0	0.4	970.5 (830.0, 1168.4)	448.7 – 2117.8	
Angiotensinogen (634.9 / 956.6)	*	34.6 (25.7, 45.1)	5.0 – 203.9	6.8	31.1 (21.4, 42.8)	5.0 – 142.4	5.6
ApoA1 (700.8 / 1023.5)	*	419.0 (337.9, 510.4)	5.0 – 907.8	0.4	317.6 (249.3, 399.0)	44.8 – 755.4	
ApoA-II-pre (486.8 / 546.4)	*	80.2 (65.2, 97.3)	5.0 – 196.6	0.4	63.9 (49.3, 77.8)	5.0 – 138.9	0.3
ApoA-Q1 (626.8 / 422.2)	*	127.9 (104.5, 156.8)	5.0 – 315.3	0.4	109.1 (87.8, 133.5)	30.5 – 233.1	
ApoB-100 (655.1 / 975.4)	*	50.6 (38.2, 68.7)	5.0 – 294.9	2.2	Intraclass correlation < 0.4		2.6
ApoB-Q1 (640.8 / 838.4)	*	93.6 (73.3, 117.2)	5.0 – 289.3	0.5	94.4 (70.6, 116.8)	5.0 – 242.2	0.3
ApoC-I (516.8 / 466.2)	*	135.9 (102.3, 178.8)	5.0 – 906.0	0.4	94.5 (67.7, 140.2)	15.7 – 533.9	
ApoC-II (745.1 / 1149.7)	*	Intraclass correlation < 0.4		63.3	Intraclass correlation < 0.4		60.1
ApoC-III (598.8 / 854.4)	*	297.4 (231.7, 381.6)	5.0 – 1258.3	0.4	220.7 (170.9, 281.5)	48.2 – 1034.2	
ApoE-Q (484.8 / 588.3)	*	72.1 (58.4, 91.5)	5.0 – 250.1	0.6	55.3 (42.7, 71.1)	5.0 – 164.7	0.6
ApoL1 (637.8 / 932.5)	*	24.5 (5.0, 36.3)	5.0 – 90.7	26.8	5.0 (5.0, 5.0)	5.0 – 43.0	93.5
Beta-2-glycoprotein I (511.8 / 751.4)	*	46.4 (37.0, 58.3)	5.0 – 132.5	1.7	48.3 (39.1, 59.9)	5.0 – 131.9	0.3
Beta-2-microglobulin (575.0 / 920.3)	*	847.0 (569.5, 1162.8)	5.0 – 2247.0	0.4	815.6 (512.3, 1109.5)	184.9 – 1922.8	
Bone morphogenetic protein 5 (723.5 / 577.3)	*	204.5 (174.8, 241.9)	5.0 – 618.1	0.4	Intraclass correlation < 0.4		
BPI fold-containing family A member 2 (481.3 / 500.4)	*	2213.5 (1982.9, 2475.4)	5.0 – 4531.0	0.4	1963.1 (1724.9, 2188.5)	388.0 – 4388.5	
C1QTNF1 (547.7 / 707.4)	*	Intraclass correlation < 0.4		0.5	162.7 (110.7, 196.1)	24.0 – 411.8	
Caeruloplasmin (602.3 / 695.3)	*	29.8 (21.4, 41.4)	5.0 – 99.8	9.5	Intraclass correlation < 0.4		9.7
Caeruloplasmin (760.1 / 1059.8)	*	Intraclass correlation < 0.4		8.1	Intraclass correlation < 0.4		6.2
Caeruloplasmin (760.6 / 931.4)	*	72.8 (54.0, 92.5)	5.0 – 250.2	0.6	64.3 (48.1, 85.3)	5.0 – 271.1	0.3
Carboxypeptidase A4 (492.9 / 399.3)	*	168.3 (135.5, 210.3)	5.0 – 558.0	0.4	119.8 (88.0, 166.9)	5.0 – 343.2	0.6
Carboxypeptidase M (501.8 / 874.5)	*	49.0 (39.5, 58.4)	5.0 – 101.2	0.9	Intraclass correlation < 0.4		2.1
CART std (576.2 / 705.3)	*	63.7 (49.9, 77.5)	5.0 – 151.3	0.6	52.4 (39.2, 64.8)	5.0 – 122.0	0.3
Cathepsin S (359.2 / 717.4)	*	33.3 (23.9, 49.2)	5.0 – 135.0	5.5	Intraclass correlation < 0.4		15.0
C-C motif chemokine 14 (476.2 / 666.3)	*	267.1 (215.2, 326.4)	5.0 – 815.5	0.4	Intraclass correlation < 0.4		
Cellular repressor of E1A-stimulated genes 1 (575.8 / 704.4)	*	186.9 (164.3, 211.4)	5.0 – 341.3	0.4	159.8 (141.1, 180.4)	69.4 – 278.5	
Chromogranin A (488.2 / 775.4)	*	36.9 (25.6, 46.9)	5.0 – 123.5	10.7	34.9 (25.1, 48.8)	5.0 – 119.6	11.7
Chromogranin B (496.2 / 764.3)	*	55.4 (42.1, 69.9)	5.0 – 215.9	2.2	50.5 (40.0, 63.2)	5.0 – 117.2	0.9
Chromogranin B (579.3 / 815.4)	*	70.1 (55.4, 85.0)	5.0 – 169.3	0.6	Intraclass correlation < 0.4		0.3
Chromogranin B (1073.5 / 822.4)	*	Intraclass correlation < 0.4		0.8	Intraclass correlation < 0.4		6.2
Clusterin (697.5 / 922.4)	*	74.2 (59.2, 91.8)	5.0 – 287.0	0.5	Intraclass correlation < 0.4		
Clusterin isoform1 (559.3 / 903.5)	*	73.2 (58.5, 89.7)	5.0 – 188.5	0.5	69.9 (53.0, 88.7)	19.3 – 162.0	
Coagulation factor IX pre (626.3 / 792.4)	*	Intraclass correlation < 0.4		17.6	Intraclass correlation < 0.4		16.1
Coagulation factor V (555.8 / 898.6)	*	329.0 (215.0, 432.5)	2.5 – 778.0	5.0	297.9 (209.0, 359.8)	5.0 – 675.1	3.8

ESM Table 2: Summary characteristics of all biomarkers measured in the study *(continued)*

		SDRNT1BIO			FinnDiane		
Biomarker	Units	Median (IQR)	Range	Below	Median (IQR)	Range	Below
Coagulation factor V (659.0 / 925.5)	*	Intraclass correlation < 0.4		63.5	Affected by storage conditions		68.0
Coagulation factor XIIa HC (442.3 / 685.4)	*	123.2 (95.6, 154.9)	5.0 – 270.8	0.4	108.8 (83.6, 133.1)	5.0 – 278.9	0.3
Complement C1q subcomponent subunit A (420.0 / 244.0)	*	224.6 (178.2, 271.4)	5.0 – 782.7	0.4	203.1 (160.3, 253.4)	63.5 – 547.0	
Complement C3 (501.8 / 731.4)	*	64.4 (52.8, 78.2)	5.0 – 154.1	0.8	Intraclass correlation < 0.4		0.3
Complement C3 (673.4 / 646.4)	*	130.5 (100.1, 155.2)	5.0 – 295.8	0.4	113.0 (89.9, 135.4)	36.8 – 241.7	
Complement C4 beta (557.8 / 629.4)	*	109.4 (88.0, 138.2)	5.0 – 276.5	0.4	100.3 (84.9, 128.7)	29.8 – 218.0	
Complement C6 (612.8 / 788.4)	*	30.8 (5.0, 47.5)	5.0 – 105.2	32.6	Intraclass correlation < 0.4		22.0
Complement C7 (550.7 / 577.3)	*	Intraclass correlation < 0.4		76.2	Intraclass correlation < 0.4		58.4
Complement C8 alpha (501.7 / 726.3)	*	Intraclass correlation < 0.4		13.4	27.9 (20.6, 37.1)	5.0 – 106.7	11.7
Complement C8 beta (694.9 / 942.6)	*	453.0 (360.8, 541.4)	5.0 – 1128.7	0.4	303.0 (249.4, 380.3)	131.4 – 809.6	
Complement C8 beta (809.9 / 1091.5)	*	59.2 (38.7, 85.5)	5.0 – 228.9	7.0	49.5 (23.4, 73.5)	5.0 – 190.1	8.8
Complement C8 gamma (810.9 / 836.4)	*	Intraclass correlation < 0.4		43.4	Intraclass correlation < 0.4		55.7
Complement factor B (578.3 / 671.4)	*	235.1 (187.8, 292.0)	5.0 – 610.8	0.4	219.8 (173.8, 292.2)	74.8 – 557.0	
Complement Factor I (1007.5 / 1023.5)	*	171.5 (95.0, 386.6)	5.0 – 741.5	0.5	Intraclass correlation < 0.4		0.3
Corticosteroid-binding globulin (539.1 / 517.3)	*	Intraclass correlation < 0.4		1.0	Intraclass correlation < 0.4		0.3
Corticotropin-releasing factor-binding protein (617.8 / 674.3)	*	Intraclass correlation < 0.4		0.8	Intraclass correlation < 0.4		0.3
C-reactive protein (564.8 / 609.4)	*	93.0 (74.9, 116.1)	5.0 – 234.1	0.8	Intraclass correlation < 0.4		1.8
C-reactive protein (569.7 / 829.3)	*	Intraclass correlation < 0.4		28.1	Intraclass correlation < 0.4		24.9
C-reactive protein iso1 (696.9 / 1035.6)	*	Intraclass correlation < 0.4		19.8	Intraclass correlation < 0.4		14.1
Cystatin C (685.0 / 412.1)	*	Intraclass correlation < 0.4		0.6	Intraclass correlation < 0.4		0.9
E-selectin (613.8 / 982.5)	*	143.4 (117.5, 178.3)	5.0 – 385.5	0.4	127.0 (102.7, 154.0)	58.3 – 381.4	
Extracellular glycoprotein lacritin (481.3 / 501.3)	*	2040.0 (1845.5, 2283.0)	5.0 – 3990.0	0.4	1797.4 (1562.7, 2034.6)	400.9 – 3403.0	
Fas (TNFRSF6) associated factor 1 (607.8 / 786.5)	*	340.0 (242.1, 451.3)	5.0 – 1898.5	1.4	306.5 (227.3, 405.6)	5.0 – 1025.4	1.2
Ferritin HC (673.3 / 1035.5)	*	Intraclass correlation < 0.4		0.4	6138.0 (3942.0, 7832.5)	1194.6 – 17092.4	
Fetuin-B (498.4 / 467.3)	*	Intraclass correlation < 0.4		27.5	Intraclass correlation < 0.4		19.4
Fibronectin iso1 (536.3 / 680.4)	*	66.4 (45.0, 101.5)	5.0 – 980.4	1.2	39.8 (24.6, 63.5)	5.0 – 408.3	6.2
Fractalkine (612.8 / 977.5)	*	49.5 (34.9, 67.1)	2.5 – 157.2	8.1	44.7 (31.0, 59.7)	5.0 – 168.0	7.9
Haptoglobin (490.5 / 562.6)	*	157.1 (110.6, 216.2)	5.0 – 711.9	0.5	138.9 (95.8, 190.7)	25.8 – 590.8	
Haptoglobin (599.4 / 658.4)	*	145.8 (110.7, 184.4)	5.0 – 598.0	0.9	120.1 (78.5, 170.0)	5.0 – 537.6	1.2
Haptoglobin (602.8 / 803.7)	*	123.4 (94.7, 161.4)	5.0 – 514.9	1.2	105.7 (80.7, 135.0)	11.6 – 372.9	
Haptoglobin beta chain (490.8 / 562.3)	*	Intraclass correlation < 0.4		0.5	789.3 (537.7, 1034.9)	114.5 – 3028.0	
Heat shock protein 60kDa (456.8 / 515.4)	*	72.6 (56.3, 94.2)	5.0 – 209.5	1.1	65.4 (53.1, 80.5)	5.0 – 178.3	1.2
Heat shock protein 90alpha iso1 (675.4 / 921.5)	*	5.0 (5.0, 5.0)	5.0 – 60.2	73.4	Intraclass correlation < 0.4		72.7
Hemopexin (571.5 / 650.4)	*	204.0 (168.0, 252.0)	5.0 – 505.0	0.4	176.3 (146.6, 212.2)	83.9 – 365.2	
Hemopexin (610.8 / 959.6)	*	929.0 (808.5, 1054.5)	5.0 – 1913.0	0.4	802.0 (651.7, 933.8)	354.0 – 1559.6	
Heparin cofactor II (514.8 / 814.4)	*	81.1 (67.0, 98.0)	5.0 – 191.5	0.4	76.0 (61.4, 90.2)	33.9 – 203.4	
Hyaluronan-binding protein 2 (575.2 / 901.5)	*	197.6 (169.7, 226.7)	5.0 – 335.4	0.4	172.2 (145.4, 195.1)	69.7 – 321.7	
Ig gamma-1 chain C region (594.0 / 698.7)	*	91.5 (65.2, 157.2)	5.0 – 355.5	0.4	Intraclass correlation < 0.4		
Immunoglobulin J chain (695.5 / 971.4)	*	40.7 (25.4, 58.6)	5.0 – 141.1	16.4	38.4 (23.6, 51.6)	5.0 – 134.8	8.2
Immunoglobulin lambda-like polypeptide 5 (421.3 / 429.2)	*	97.5 (79.4, 117.5)	5.0 – 239.0	0.4	91.1 (74.0, 110.4)	5.0 – 221.6	0.3
Inhibin beta B chain (594.8 / 419.3)	*	159.6 (130.3, 202.4)	5.0 – 625.4	0.4	129.2 (96.2, 163.6)	31.4 – 1012.4	
Insulin-like growth factor-binding protein2 (530.6 / 785.4)	*	20.4 (13.8, 27.2)	5.0 – 77.1	15.6	Intraclass correlation < 0.4		16.4
Inter-alpha-trypsin inhibitor HC (579.3 / 902.5)	*	143.3 (119.6, 166.2)	5.0 – 275.1	0.4	111.7 (90.1, 134.8)	44.1 – 231.5	
Interleukin-26 (575.2 / 512.3)	*	104.2 (88.1, 122.2)	5.0 – 224.1	0.4	85.7 (73.2, 103.4)	42.4 – 189.0	
Kallistatin (643.4 / 971.6)	*	340.2 (289.4, 407.1)	5.0 – 857.7	0.4	Affected by storage conditions		48.4
Kininogen-1 (626.3 / 173.1)	*	Intraclass correlation < 0.4		0.4	Intraclass correlation < 0.4		
Leucine-rich alpha-2-glycoprotein (590.5 / 725.4)	*	141.6 (115.9, 173.6)	5.0 – 357.2	0.4	128.1 (102.5, 159.6)	43.7 – 2840.0	
MPO (575.3 / 939.4)	*	5.0 (5.0, 34.6)	5.0 – 168.1	55.8	Intraclass correlation < 0.4		33.4
Osteopontin isoA (694.3 / 853.4)	*	241.2 (191.0, 296.7)	5.0 – 556.3	0.4	163.5 (130.9, 206.0)	66.2 – 347.1	
Pericentriolar material 1 (526.3 / 825.5)	*	49.7 (39.0, 64.4)	5.0 – 167.2	2.1	48.2 (37.0, 61.1)	5.0 – 117.3	2.1
Peroxidase (492.6 / 703.3)	*	30.3 (21.3, 42.9)	5.0 – 93.5	9.8	26.8 (18.0, 38.1)	5.0 – 100.3	16.1
Peroxiredoxin 2 (489.8 / 735.4)	*	140.0 (107.2, 186.0)	5.0 – 617.5	0.6	115.8 (86.0, 160.1)	5.0 – 344.8	0.3

ESM Table 2: Summary characteristics of all biomarkers measured in the study *(continued)*

		SDRNT1BIO			FinnDiane		
Biomarker	Units	Median (IQR)	Range	Below	Median (IQR)	Range	Below
Phosphatidylcholine-sterol acyltransferase (693.7 / 941.5)	*	28.3 (17.5, 44.4)	5.0 – 291.7	18.6	26.7 (12.6, 53.3)	5.0 – 146.5	20.5
Plasma glutamate carboxypeptidase (577.3 / 743.3)	*	Intraclass correlation < 0.4		48.4	Intraclass correlation < 0.4		41.9
Plasma protease C1 inhibitor (633.0 / 1049.6)	*	56.5 (44.9, 71.5)	5.0 – 288.6	1.0	Intraclass correlation < 0.4		0.3
Plasminogen (438.3 / 502.3)	*	28.6 (20.7, 36.4)	5.0 – 85.2	11.5	Intraclass correlation < 0.4		8.2
Plasminogen (438.3 / 615.3)	*	310.0 (261.5, 379.5)	5.0 – 2073.0	0.4	294.1 (247.7, 347.9)	118.7 – 1190.0	
Plasminogen (570.8 / 699.4)	*	92.0 (75.0, 111.0)	5.0 – 225.1	0.5	84.6 (72.6, 103.9)	32.9 – 195.2	
Polymerase (RNA) II polypeptide D (425.2 / 635.3)	*	36.9 (26.6, 48.0)	5.0 – 137.0	5.1	33.0 (22.9, 45.3)	5.0 – 131.6	7.0
PON1 Serum paraoxonase/arylesterase 2 (942.5 / 472.3)	*	234.0 (174.0, 317.0)	5.0 – 869.0	0.4	250.0 (174.6, 372.9)	52.7 – 666.8	
Prothrombin (626.3 / 679.4)	*	45.4 (34.9, 57.6)	5.0 – 117.2	1.9	Intraclass correlation < 0.4		2.1
Prothrombin (626.3 / 879.5)	*	39.0 (30.0, 50.2)	5.0 – 108.7	3.6	Intraclass correlation < 0.4		2.6
P-selectin (500.2 / 492.3)	*	18.1 (5.0, 33.2)	5.0 – 456.5	31.9	17.4 (5.0, 134.6)	5.0 – 366.2	32.8
P-selectin (712.4 / 876.5)	*	Intraclass correlation < 0.4		15.9	Intraclass correlation < 0.4		7.9
RBP1 (367.2 / 505.3)	*	Intraclass correlation < 0.4		55.2	5.0 (5.0, 10.6)	5.0 – 30.9	70.4
Retinal binding protein 2 (575.8 / 695.3)	*	4156.2 (3666.6, 4584.2)	5.0 – 6884.7	0.4	3571.3 (3086.9, 3986.7)	1725.1 – 6580.2	
RBP4 (481.2 / 833.4)	*	Intraclass correlation < 0.4		0.4	136.9 (101.4, 183.0)	29.1 – 333.8	
RBP (583.3 / 669.5)	*	59.3 (44.2, 78.6)	5.0 – 219.6	0.9	67.4 (53.2, 91.8)	12.6 – 230.4	
Renin isoform1 (520.8 / 502.3)	*	86.1 (59.4, 120.2)	5.0 – 321.5	0.6	67.2 (34.5, 105.1)	5.0 – 224.7	0.9
Renin isoform1 (610.3 / 959.4)	*	41.7 (24.3, 107.0)	5.0 – 474.3	7.1	Intraclass correlation < 0.4		5.3
Reticulon-4 isoform1 (618.8 / 894.5)	*	13.6 (5.0, 21.5)	2.5 – 86.9	34.5	Intraclass correlation < 0.4		30.5
sCD40L (471.2 / 837.4)	*	122.8 (97.7, 152.2)	5.0 – 351.5	0.4	Intraclass correlation < 0.4		
Serotransferrin (489.9 / 735.4)	*	164.0 (129.8, 215.1)	5.0 – 602.5	0.4	133.3 (100.5, 174.7)	47.2 – 366.0	
Serotransferrin (625.5 / 675.4)	*	330.4 (255.4, 419.8)	5.0 – 866.0	0.5	265.6 (210.1, 335.8)	72.6 – 696.5	
Serum Amyloid A (739.4 / 1151.6)	*	Intraclass correlation < 0.4		21.1	Intraclass correlation < 0.4		21.7
Serum amyloid P-component (578.8 / 508.3)	*	Intraclass correlation < 0.4		0.9	Intraclass correlation < 0.4		0.3
Somatostatin receptor type 2-P30874 (575.8 / 674.4)	*	97.0 (81.4, 114.5)	5.0 – 207.9	0.4	81.5 (68.7, 98.2)	31.5 – 154.5	
TAO kinase 1 (645.3 / 804.4)	*	78.8 (58.8, 98.2)	5.0 – 200.8	0.6	60.9 (48.6, 77.8)	5.0 – 160.5	0.6
Thyroxine binding globulin (530.8 / 244.1)	*	51.0 (28.1, 111.4)	5.0 – 527.5	7.9	Intraclass correlation < 0.4		6.7
Thyroxine-binding globulin (801.1 / 993.9)	*	77.2 (56.3, 99.5)	5.0 – 210.8	1.4	Intraclass correlation < 0.4		34.9
TNF alpha (691.9 / 1067.7)	*	67.1 (54.6, 83.2)	5.0 – 204.1	1.2	Intraclass correlation < 0.4		0.9
TNFRSF9 (409.7 / 519.3)	*	572.8 (421.8, 715.1)	5.0 – 4890.7	0.4	598.7 (350.3, 820.6)	101.4 – 3230.4	
Transferrin (489.7 / 735.4)	*	138.9 (106.1, 179.0)	5.0 – 619.0	0.5	109.9 (84.7, 157.2)	29.5 – 409.3	
Transferrin (598.3 / 993.5)	*	72.3 (56.2, 88.8)	5.0 – 180.4	1.3	58.5 (46.2, 73.8)	5.0 – 183.7	0.9
Transferrin (739.9 / 1184.6)	*	Intraclass correlation < 0.4		0.4	271.4 (168.1, 361.2)	37.1 – 832.9	
Troponin I (581.8 / 935.4)	*	Intraclass correlation < 0.4		0.8	91.5 (70.8, 113.7)	25.3 – 251.3	
Ubiquinol-cytochrome c reductase core protein II (547.3 / 639.3)	*	Intraclass correlation < 0.4		0.4	1069.0 (658.1, 1377.4)	162.5 – 2569.7	
Vitamin K-dependent protein C (516.3 / 603.3)	*	62.3 (49.9, 74.9)	5.0 – 167.3	0.8	Intraclass correlation < 0.4		30.8
Vit D binding (628.2 / 691.3)	*	70.0 (52.9, 87.8)	5.0 – 209.0	1.2	Intraclass correlation < 0.4		53.1
Vitronectin (711.8 / 647.3)	*	98.7 (82.6, 119.5)	5.0 – 320.4	0.4	86.9 (74.7, 104.5)	38.2 – 200.5	
Von Willebrand factor (500.8 / 788.4)	*	57.8 (46.4, 70.0)	5.0 – 120.9	1.5	67.9 (57.1, 81.2)	24.6 – 138.5	
WD repeat domain 67-iso1 (487.8 / 604.3)	*	44.4 (33.9, 55.6)	5.0 – 130.0	4.7	44.4 (33.3, 55.5)	5.0 – 119.6	3.2
Zinc-alpha-2-glycoprotein (697.8 / 789.4)	*	51.4 (34.0, 68.3)	5.0 – 172.6	11.5	Intraclass correlation < 0.4		8.5

Note:
Biomarkers that were not analyzed report the reason for their exclusion from the analysis
An * indicates that the value is a ratio of the biomarker to a stable isotope of albumin T6
Metabolites with “[conf]” in their name represent confidence transitions
“Below” reports the percentage of observations with levels below the detection threshold

ESM Table 3: Associations of each biomarker (considered separately) with achieved eGFR from linear regression models adjusted for age, sex, duration of diabetes, study day eGFR, length of follow-up and ACR category

	SDRNT1BIO		FinnDiane	
Biomarker	Coefficient (95% CI)	*p*	Coefficient (95% CI)	*p*
**Luminex proteins**				
CD27 antigen	-0.22 (-0.27, -0.17)	2E-16	-0.34 (-0.46, -0.23)	9E-09
Kidney Injury Molecule-1	-0.16 (-0.21, -0.11)	6E-10	-0.27 (-0.37, -0.18)	6E-08
Alpha-1-Microglobulin	-0.18 (-0.24, -0.13)	2E-11	-0.26 (-0.36, -0.16)	4E-07
Beta-2-Microglobulin	-0.22 (-0.27, -0.17)	1E-15	-0.26 (-0.37, -0.14)	1E-05
Cystatin-C	-0.23 (-0.29, -0.18)	3E-15	-0.20 (-0.31, -0.09)	3E-04
Thrombomodulin	-0.19 (-0.24, -0.14)	3E-12	-0.22 (-0.32, -0.11)	5E-05
Tumor Necrosis Factor Receptor 1	-0.17 (-0.22, -0.12)	1E-10	-0.20 (-0.30, -0.09)	3E-04
Osteopontin	-0.12 (-0.17, -0.07)	1E-06	-0.18 (-0.28, -0.08)	7E-04
Interleukin-2 receptor alpha	-0.16 (-0.20, -0.11)	2E-10	-0.11 (-0.21, -0.01)	3E-02
Fibroblast Growth Factor 21	-0.11 (-0.15, -0.06)	5E-06	-0.15 (-0.25, -0.06)	1E-03
N-terminal prohormone of brain natriuretic peptide	-0.12 (-0.17, -0.07)	6E-06	0.00 (-0.10, 0.09)	9E-01
Tamm-Horsfall Urinary Glycoprotein	0.11 (0.06, 0.15)	3E-05	0.09 (-0.01, 0.18)	8E-02
Trefoil Factor 3	-0.11 (-0.16, -0.06)	2E-05	*Not tested*	
**LC-MSMS metabolites**				
Free Sialic acid	-0.20 (-0.26, -0.15)	2E-12	-0.21 (-0.33, -0.08)	1E-03
SDMA	-0.15 (-0.21, -0.09)	2E-07	-0.21 (-0.33, -0.09)	6E-04
Tryptophan/Kynurenine	0.17 (0.12, 0.22)	3E-10	0.08 (-0.03, 0.18)	2E-01
C5DC (Glutaryl) Carnitine	-0.11 (-0.16, -0.06)	4E-05	-0.07 (-0.18, 0.04)	2E-01
Free Cystine	-0.11 (-0.16, -0.05)	7E-05	*Not tested*	
Tryptophan	0.11 (0.07, 0.16)	2E-06	*Not tested*	
Methionine	0.10 (0.05, 0.14)	3E-05	*Not tested*	
**LC-MSMS tryptic peptides**				
Albumin T70 (501.2 / 587.5)	0.10 (0.05, 0.14)	3E-05	-0.01 (-0.10, 0.08)	8E-01
Chromogranin A (488.2 / 775.4)	-0.10 (-0.14, -0.05)	3E-05	0.00 (-0.09, 0.09)	1E+00

Regression coefficients are per unit of standard deviation of gaussianised biomarker
We report median and interquartile range (IQR) for continuous variables, and frequency for categorical variables

ESM Table 4: Associations of each biomarker (considered separately) with achieved eGFR from linear regression models adjusted for full clinical covariates

	SDRNT1BIO		FinnDiane	
Biomarker	Coefficient (95% CI)	*p*	Coefficient (95% CI)	*p*
**Luminex proteins**				
CD27 antigen	-0.17 (-0.22, -0.12)	9E-12	-0.32 (-0.43, -0.21)	4E-08
Beta-2-Microglobulin	-0.16 (-0.21, -0.11)	2E-09	-0.26 (-0.37, -0.15)	9E-06
Alpha-1-Microglobulin	-0.14 (-0.19, -0.09)	1E-07	-0.23 (-0.32, -0.13)	5E-06
Kidney Injury Molecule-1	-0.14 (-0.19, -0.10)	6E-09	-0.23 (-0.33, -0.13)	6E-06
Cystatin-C	-0.17 (-0.22, -0.11)	2E-09	-0.22 (-0.33, -0.12)	5E-05
Osteopontin	-0.09 (-0.14, -0.05)	9E-05	-0.19 (-0.29, -0.09)	2E-04
Thrombomodulin	-0.14 (-0.18, -0.09)	1E-07	-0.19 (-0.29, -0.09)	2E-04
Tumor Necrosis Factor Receptor 1	-0.12 (-0.17, -0.08)	2E-07	-0.19 (-0.29, -0.09)	3E-04
Interleukin-2 receptor alpha	-0.11 (-0.16, -0.07)	1E-06	-0.11 (-0.21, -0.02)	2E-02
**LC-MSMS metabolites**				
SDMA	-0.11 (-0.16, -0.06)	3E-05	-0.21 (-0.33, -0.10)	3E-04
Free Sialic acid	-0.14 (-0.19, -0.08)	6E-07	-0.18 (-0.30, -0.05)	6E-03
Tryptophan/Kynurenine	0.14 (0.09, 0.18)	6E-08	0.11 (0.00, 0.21)	5E-02
Tryptophan	0.09 (0.04, 0.13)	6E-05	*Not tested*	

Regression coefficients are per unit of standard deviation of gaussianised biomarker
We report median and interquartile range (IQR) for continuous variables, and frequency for categorical variables
Full clinical covariates: age, sex, diabetes duration, study day eGFR, length of follow-up, categorical ACR, BMI, DBP, SBP, HbA1c, HDL-cholesterol, total cholesterol, smoking status, weighted average of historical eGFR

ESM Table 5: Ranking of the top biomarkers for prediction of achieved eGFR produced by the projective selection approach from linear regression models adjusted for age, sex, duration of diabetes, study day eGFR and length of follow-up

#	SDRNT1BIO	FinnDiane
**Luminex**		
1	CD27 antigen	CD27 antigen
2	Kidney Injury Molecule-1	Kidney Injury Molecule-1
3	Cystatin-C	Tamm-Horsfall Urinary Glycoprotein
4	Tamm-Horsfall Urinary Glycoprotein	Alpha-1-Microglobulin
5	Tumor Necrosis Factor Receptor 1	Fibroblast Growth Factor 23
6	Thrombomodulin	Osteopontin
7	N-terminal prohormone of brain natriuretic peptide	Tissue Inhibitor of Metalloproteinases 1
8	Alpha-1-Microglobulin	Latency-Associated-Peptide
9	Osteopontin	Thrombomodulin
10	Lectin-Like Oxidized LDL Receptor 1	Growth-Regulated alpha protein
**LC-MSMS**		
1	Free Sialic acid	Free Sialic acid
2	Albumin T70 (501.2 / 587.5)	Beta-2-microglobulin (575.0 / 920.3)
3	Tryptophan/Kynurenine	Heat shock protein 60kDa (456.8 / 515.4)
4	Chromogranin A (488.2 / 775.4)	SDMA
5	Hydroxyproline	Retinal binding protein 2 (575.8 / 695.3)
6	Osteopontin isoA (694.3 / 853.4)	Xanthine
7	Extracellular glycoprotein lacritin (481.3 / 501.3)	Alpha-2-macroglobulin (697.8 / 737.4)
8	SDMA	Hexitol
9	ApoC-III (598.8 / 854.4)	Glycine/Valine
10	Tyrosine	Alpha-fetoprotein (490.7 / 833.3)
**Both platforms**		
1	CD27 antigen	CD27 antigen
2	Kidney Injury Molecule-1	Kidney Injury Molecule-1
3	Free Sialic acid	Beta-2-microglobulin (575.0 / 920.3)
4	Angiotensin II (349.8 / 136.1)	SDMA
5	Thrombomodulin	Histidine
6	Tryptophan/Kynurenine	Alpha-1-Microglobulin
7	3-Methyl-histidine	Serotransferrin (625.5 / 675.4)
8	Histidine	Fibronectin iso1 (536.3 / 680.4)
9	Citrulline	Free Sialic acid
10	Heparin cofactor II (514.8 / 814.4)	Thrombomodulin

Physicians and nurses at health care centers participating in the collection of FinnDiane patients.

FinnDiane Study Centers	Physicians and nurses
Anjalankoski Health Centre	S. Koivula, T. Uggeldahl
Central Finland Central Hospital, Jyväskylä	T. Forslund, A. Halonen, A. Koistinen, P. Koskiaho, M. Laukkanen, J. Saltevo, M. Tiihonen
Central Hospital of Åland Islands, Mariehamn	M. Forsen, H. Granlund, A-C. Jonsson, B. Nyroos
Central Hospital of Kanta-Häme, Hämeenlinna	P. Kinnunen, A. Orvola, T. Salonen, A. Vähänen
Central Hospital of Länsi-Pohja, Kemi	H. Laukkanen, P. Nyländen, A. Sademies
Central Ostrabothnian Hospital District, Kokkola	S. Anderson, B. Asplund, U. Byskata, P. Liedes, M. Kuusela, T. Virkkala
City of Espoo Health Centre	
Espoonlahti	A. Nikkola, E. Ritola
Tapiola	M. Niska, H. Saarinen
Samaria	E. Oukko-Ruponen, T. Virtanen
Viherlaakso	A. Lyytinen
City of Helsinki Health Centre	
Puistola	H. Kari, T. Simonen
Suutarila	A. Kaprio, J. Kärkkäinen, B. Rantaeskola
Töölö	P. Kääriäinen, J. Haaga, A-L. Pietiläinen
City of Hyvinkää Health Centre	S. Klemetti, T. Nyandoto, E. Rontu, S. Satuli-Autere
City of Vantaa Health Centre	
Korso	R. Toivonen, H. Virtanen
Länsimäki	R. Ahonen, M. Ivaska-Suomela, A. Jauhiainen
Martinlaakso	M. Laine, T. Pellonpää, R. Puranen
Myyrmäki	A. Airas, J. Laakso, K. Rautavaara
Rekola	M. Erola, E. Jatkola
Tikkurila	R. Lönnblad, A. Malm, J. Mäkelä, E. Rautamo
Heinola Health Centre	P. Hentunen, J. Lagerstam
Helsinki University Central Hospital, Department of Medicine, Division of Nephrology	A. Ahola, J. Fagerudd, M. Feodoroff, D. Gordin, O. Heikkilä, K Hietala, L. Kyllönen, J. Kytö, S. Lindh, K. Pettersson-Fernholm, M. Rosengård-Bärlund, M. Rönnback, A. Sandelin, A-R Salonen, L. Salovaara, L. Thorn, J. Tuomikangas, T. Vesisenaho, J. Wadén
Herttoniemi Hospital, Helsinki	V. Sipilä
Hospital of Lounais-Häme, Forssa	T. Kalliomäki, J. Koskelainen, R. Nikkanen, N. Savolainen, H. Sulonen, E. Valtonen
Iisalmi Hospital	E. Toivanen
Jokilaakso Hospital, Jämsä	A. Parta, I. Pirttiniemi
Jorvi Hospital, Helsinki University Central Hospital	S. Aranko, S. Ervasti, R. Kauppinen-Mäkelin, A. Kuusisto, T. Leppälä, K. Nikkilä, L. Pekkonen
Jyväskylä Health Centre, Kyllö	K. Nuorva, M. Tiihonen
Kainuu Central Hospital, Kajaani	S. Jokelainen, P. Kemppainen, A-M. Mankinen, M. Sankari
Kerava Health Centre	H. Stuckey, P. Suominen
Kirkkonummi Health Centre	A. Lappalainen, M. Liimatainen, J. Santaholma
Kivelä Hospital, Helsinki	A. Aimolahti, E. Huovinen
Koskela Hospital, Helsinki	V. Ilkka, M. Lehtimäki
Kotka Heath Centre	E. Pälikkö-Kontinen, A. Vanhanen
Kouvola Health Centre	E. Koskinen, T. Siitonen

FinnDiane Study Centers	Physicians and nurses
Kuopio University Hospital	E. Huttunen, R. Ikäheimo, P. Karhapää, P. Kekäläinen, M. Laakso, T. Lakka, E. Lampainen, L. Moilanen, L. Niskanen, U. Tuovinen, I. Vauhkonen, E. Voutilainen
Kuusamo Health Centre	T. Kääriäinen, E. Isopoussu
Kuusankoski Hospital	E. Kilkki, I. Koskinen, L. Riihelä
Laakso Hospital, Helsinki	T. Meriläinen, P. Poukka, R. Savolainen, N. Uhlenius
Lahti City Hospital	A. Mäkelä, M. Tanner
Lapland Central Hospital, Rovaniemi	L. Hyvärinen, S. Severinkangas, T. Tulokas
Lappeenranta Health Centre	P. Linkola, I. Pulli
Lohja Hospital	T. Granlund, M. Saari, T. Salonen
Loimaa Health Centre	A. Mäkelä, P. Eloranta
Länsi-Uusimaa Hospital, Tammisaari	I-M. Jousmaa, J. Rinne
Malmi Hospital, Helsinki	H. Lanki, S. Moilanen, M. Tilly-Kiesi
Mikkeli Central Hospital	A. Gynther, R. Manninen, P. Nironen, M. Salminen, T. Vänttinen
Mänttä Regional Hospital	I. Pirttiniemi, A-M. Hänninen
North Karelian Hospital, Joensuu	U-M. Henttula, P. Kekäläinen, M. Pietarinen, A. Rissanen, M. Voutilainen
Nurmijärvi Health Centre	A. Burgos, K. Urtamo
Oulankangas Hospital, Oulainen	E. Jokelainen, P-L. Jylkkä, E. Kaarlela, J. Vuolaspuro
Oulu Health Centre	L. Hiltunen, R. Häkkinen, S. Keinänen-Kiukaanniemi
Oulu University Hospital	R. Ikäheimo
Päijät-Häme Central Hospital	H. Haapamäki, A. Helanterä, S. Hämäläinen, V. Ilvesmäki, H. Miettinen
Palokka Health Centre	P. Sopanen, L. Welling
Pieksämäki Hospital	V. Javtsenko, M. Tamminen
Pietarsaari Hospital	M-L. Holmbäck, B. Isomaa, L. Sarelin
Pori City Hospital	P. Ahonen, P. Merensalo, K. Sävelä
Porvoo Hospital	M. Kallio, B. Rask, S. Rämö
Raahe Hospital	A. Holma, M. Honkala, A. Tuomivaara, R. Vainionpää
Rauma Hospital	K. Laine, K. Saarinen, T. Salminen
Riihimäki Hospital	P. Aalto, E. Immonen, L. Juurinen
Salo Hospital	A. Alanko, J. Lapinleimu, P. Rautio, M. Virtanen
Satakunta Central Hospital, Pori	M. Asola, M. Juhola, P. Kunelius, M-L. Lahdenmäki, P. Pääkkönen, M. Rautavirta
Savonlinna Central Hospital	E. Korpi-Hyövälti, T. Latvala, E. Leijala
South Karelia Central Hospital, Lappeenranta	T. Ensala, E. Hussi, R. Härkönen, U. Nyholm, J. Toivanen
Tampere Health Centre	A. Vaden, P. Alarotu, E. Kujansuu, H. Kirkkopelto-Jokinen, M. Helin, S. Gummerus, L. Calonius, T. Niskanen, T. Kaitala, T. Vatanen
Tampere University Hospital	I. Ala-Houhala, T. Kuningas, P. Lampinen, M. Määttä, H. Oksala, T. Oksanen, K. Salonen, H. Tauriainen, S. Tulokas
Tiirismaa Health Centre, Hollola	T. Kivelä, L, Petlin, L. Savolainen
Turku Health Centre	I. Hämäläinen, H. Virtamo, M. Vähätalo
Turku University Central Hospital	K. Breitholz, R. Eskola, K. Metsärinne, U. Pietilä, P. Saarinen, R. Tuominen, S. Äyräpää
Vaajakoski Health Centre	K. Mäkinen, P. Sopanen
Valkeakoski Regional Hospital	S. Ojanen, E. Valtonen, H. Ylönen, M. Rautiainen, T. Immonen
Vammala Regional Hospital	I. Isomäki, R. Kroneld, M. Tapiolinna-Mäkelä
Vaasa Central Hospital	S. Bergkulla, U. Hautamäki, V-A. Myllyniemi, I. Rusk

The SDRNT1BIO Study Centres

**Arran & Ayrshire**	
Ayr Hospital	Professor Andrew Collier<br>Karen Allan
University Hospital Crosshouse, Kilmarnock	Danielle Gilmour<br>Margo Henry
**Dumfries & Galloway**	
Dumfries & Galloway Royal Infirmary	Dr Fiona Green<br>Jean Bell
**Fife**	
Queen Margaret Hospital, Dunfermline	Dr John Chalmers<br>Karen Gray
**Glasgow**	
Clinical Research Facility, Western Infirmary	Dr Robert Lindsay<br>Hilary Peddie
Glasgow Royal Infirmary	Professor John Petrie
**Grampian**	
Centre of Diabetes and Endocrinology, Aberdeen	Professor Donald Pearson<br>Julie Toye
**Highlands & Islands**	
Highland Clinical Research Facility, Inverness	Professor Sandra Macrury<br>Wendy Mackinnon
**Lanarkshire**	
David Matthews Diabetes Centre, Monklands Hospital, Airdrie	Dr Sandeep Thekkepat<br>Berni Welsh<br>Linda Macliver<br>Dina Bell
**Lothian**	
Department of Diabetes, Royal Infirmary of Edinburgh	Dr Allan Patrick<br>Alison Sudworth<br>Claire Gallacher
Western General Hospital, Edinburgh	Professor John McKnight<br>Alex Clark<br>Roz Graham
NHS National Services Scotland, Edinburgh	Dr Colin Fischbacher
St Johns Hospital, Livingston	Katie Farley
**Tayside**	
Ninewells Hospital, Dundee	Professor Colin Palmer<br>Lynsey Beal<br>Sheena Pritchard
